# CARK1 mediates ABA signaling by phosphorylation of ABA receptors

**DOI:** 10.1038/s41421-018-0029-y

**Published:** 2018-06-19

**Authors:** Liang Zhang, Xiaoyi Li, Dekuan Li, Yuna Sun, Ying Li, Qin Luo, Zhibin Liu, Jianmei Wang, Xufeng Li, Hong Zhang, Zhiyong Lou, Yi Yang

**Affiliations:** 1 Key Laboratory of Bio-Resources and Eco-Environment of Ministry of Education, State Key Laboratory of Hydraulics and Mountain River Engineering, College of Life Sciences, Sichuan University, Chengdu, 610065 China; 2 National Laboratory of Macromolecules, Institute of Biophysics, Chinese Academy of Science, Beijing, 100101 China; 3 Department of Biological Sciences, Texas Tech University, Lubbock, TX 79409 USA; 4 Laboratory of Structural Biology and MOE Laboratory of Protein Science, School of Medicine and Life Sciences, Tsinghua University, Beijing, 100084 China; 5 Collaborative Innovation Center for Biotherapy, State Key Laboratory of Biotherapy and Cancer Center, West China Hospital, West China Medical School, Sichuan University, Chengdu, China

## Abstract

The function of abscisic acid (ABA) is mediated by its receptors termed RCARs/PYR1/PYLs. Modulation of ABA signaling is vital for plant growth and development. The RCAR-PP2C-SnRK2 regulatory modules have been defined as the core components in ABA signaling. However, it is still not clear whether and how the ABA receptors could be modified at the initial post-translational stage to fine-tune ABA transduction pathway. Here we identify and characterize the putative receptor-like cytoplasmic kinase (RLCK) in Arabidopsis named CARK1, which interacts with RCAR3 (PYL8) and RCAR11 (PYR1) in the manner of phosphorylation. Structural studies of CARK1 revealed the critical active site, N204, which accounts for the kinase activity and the direct interaction with RCAR3/RCAR11. CARK1 phosphorylates RCAR3/RCAR11 at one conserved threonine site, T77/T78. Our genetic analyses further demonstrated that *CARK1* positively regulates ABA-mediated physiological responses and overexpression of *CARK1* in Arabidopsis distinctly promotes the drought resistance. Moreover, the phosphor-mimic form of RCAR11 in the *cark1* mutant is able to functionally complement the ABA sensitivity. CARK1 positively regulates ABA-responsive gene expression and enhances RCAR3/RCAR11’s inhibition to Clade A PP2C. Taken together, our studies strongly support the functional significance of CARK1 in positively regulating ABA signaling via phosphorylation on RCAR3/RCAR11 in Arabidopsis.

## Introduction

The phytohormone abscisic acid (ABA) plays important roles in plant growth, development and stress responses^1, 2^. ABA’s function is mediated by ABA receptors that are a group of proteins termed PYR1/PYLs/RCARs (hereafter referred as RCARs)^3–6^. Biochemical, genetic, and structural studies have revealed the mode of action of RCARs^1–7^. RCARs contain a hydrophobic ABA binding pocket guarded by two flexible surface loops, “gate” and “latch”^8–12^. Upon ABA binding, the gate loop closes to create a surface that enables the receptor to dock and competitively inhibit the active site of Clade A protein phosphatases 2C (PP2Cs)^8–13^. This allows for the activation of SnRK2s, which directly phosphorylate ABFs/AREBs^1,2^, SLAC1^14^, and KAT1^15^ channel for ABA responses and stomatal movements, respectively. Hence, some of the downstream events are starting to be well characterized. Studies revealed that the degradation of ABA receptors was mediated either by multi-subunit CRL4-CDD E3 ligase complex and F-box E3 ligase RIFP1 or single-subunit E3 ligase RSL1^16–18^. Tyrosine nitration could also cause the degradation of ABA receptors^19^. However, other regulatory mechanisms of ABA signaling remain largely unknown.

In this study, an interactor of RCAR3 and RCAR11 was identified and named as Cytosolic ABA Receptor Kinase 1 (CARK1). CARK1 belongs to a putative Ser/Thr protein kinase RLCK VIII subfamily in Arabidopsis and displays a canonical bilobal structural architecture of protein kinase family. N204 of CARK1 is critical for its kinase activity and for the interactions between CARK1 and RCAR3/RCAR11 in vitro and in vivo. CARK1 phosphorylates RCAR3 and RCAR11 at the position T77 and T78, respectively. Functional analyses further reveal that *CARK1* positively regulates ABA-mediated physiological responses, supporting that ABA transduction pathway is fine-tuned via the phosphorylation of ABA receptors by CARK1.

## Results

### CARK1 is an interactor of RCAR3 or RCAR11

As the intracellular ABA receptors, RCARs are crucial for elucidating ABA signaling network. We used a yeast two-hybrid system to screen for interacting proteins of RCAR3. One of the candidates with no annotated function, At3g17410, was found to interact with RCAR3 and RCAR11 (Supplementary Fig. S1A-C). At3g17410 is conserved in plants and belongs to a putative Ser/Thr protein kinase RLCK VIII subfamily with 11 members in Arabidopsis (Supplementary Fig. S1D and S2B). Among them, Pti1-1/2/3/4 and MARIS (MRI) have phosphorylation activities in vitro^20–23^. Based on the cytoplasmic localization (Supplementary Fig. S4A) and its kinase activity, we therefore named At3g17410 as CARK1.

### Overall structure of CARK1-KD

**Fig. 1:**
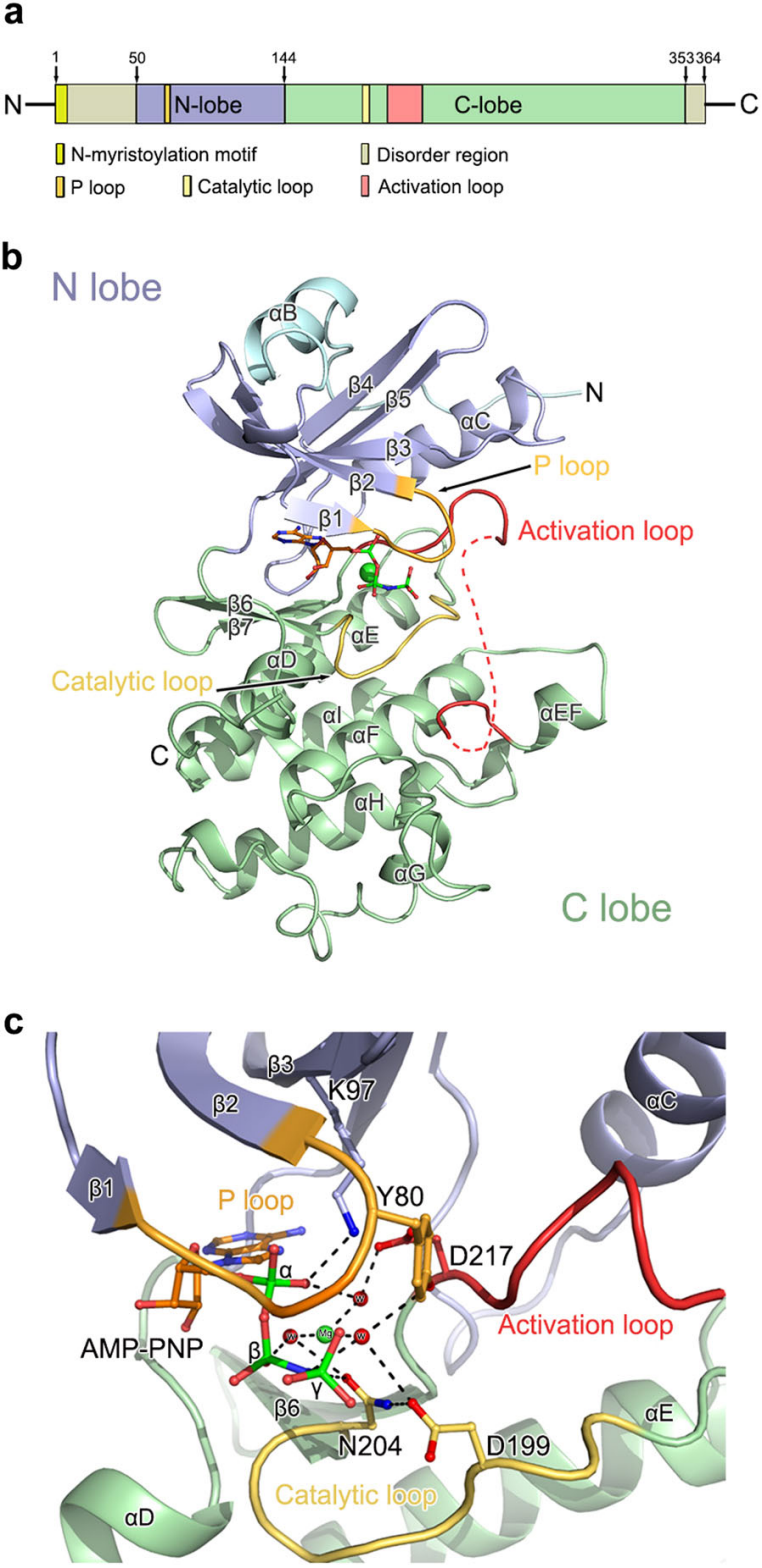
Structural analysis of CARK1-KD. **a** Schematic representations of CARK1. The amino acid residues are numbered as indicated. **b** The structure of CARK1-KD monomer A in complex with AMP-PNP. The polypeptide of CARK1-KD is shown as cartoon, the bound AMP-PNP molecule is presented as colored stick. The N-terminal extension, N-lobe, P loop, catalytic loop, activation loop, and C-lobe are colored pale cyan, light blue, bright orange, yellow-orange, TV red, and pale green, respectively. **c** Close view of CARK1 kinase catalytic site. Red spheres labeled “w” denote water molecules. Dashed lines indicate hydrogen-bonding interactions. The key residues for adenosine-binding pocket are represented as colored sticks

To study the molecular architecture of CARK1 (Fig. 1a), we first solved the crystal structure of the kinase domain of CARK1 (residues 50–353, named CARK1-KD hereafter) in complex with AMP-PNP (Fig. 1b; Supplementary Fig. S3). CARK1-KD displays a canonical bilobal structural architecture of protein kinase family^24^, consisting of a N- and a C-lobe. The N-lobe comprises a twisted five-stranded anti-parallel β-sheet with a typical single-helix αC (Fig. 1b). The C-lobe is made of predominantly with α-helical structures, containing seven α-helices and two β-strands. The activation loop (residues D217–E246) begins and ends at the highly conserved DFD (Asp-Phe-Asp) and APE (Ala-Pro-Glu) motifs in the protein kinase family^24^. In the binary complex structure of CARK1-KD-ANP, a glycine-rich nucleotide-binding loop, termed P-loop (E77 to G81), stabilizes the bound AMP-PNP through interacting with the phosphate groups of AMP-PNP molecule (Fig. 1c). In addition, two linkages with α-phosphate, including a hydrogen bond (formed with K97 in β3 strand) and a solvent-mediated hydrogen bond (formed with D217 at the activation loop) (Fig. 1c). Another interaction between hydroxyl groups of N204 and γ-phosphate emphasizes the crucial role of N204 in ATP hydrolysis, which is one of the strictly conserved residues of AtRLCK VIII family (Supplementary Fig. S2B).

### N204 of CARK1 mediates the interaction with RCAR3 or RCAR11 in vitro and in vivo

**Fig. 2:**
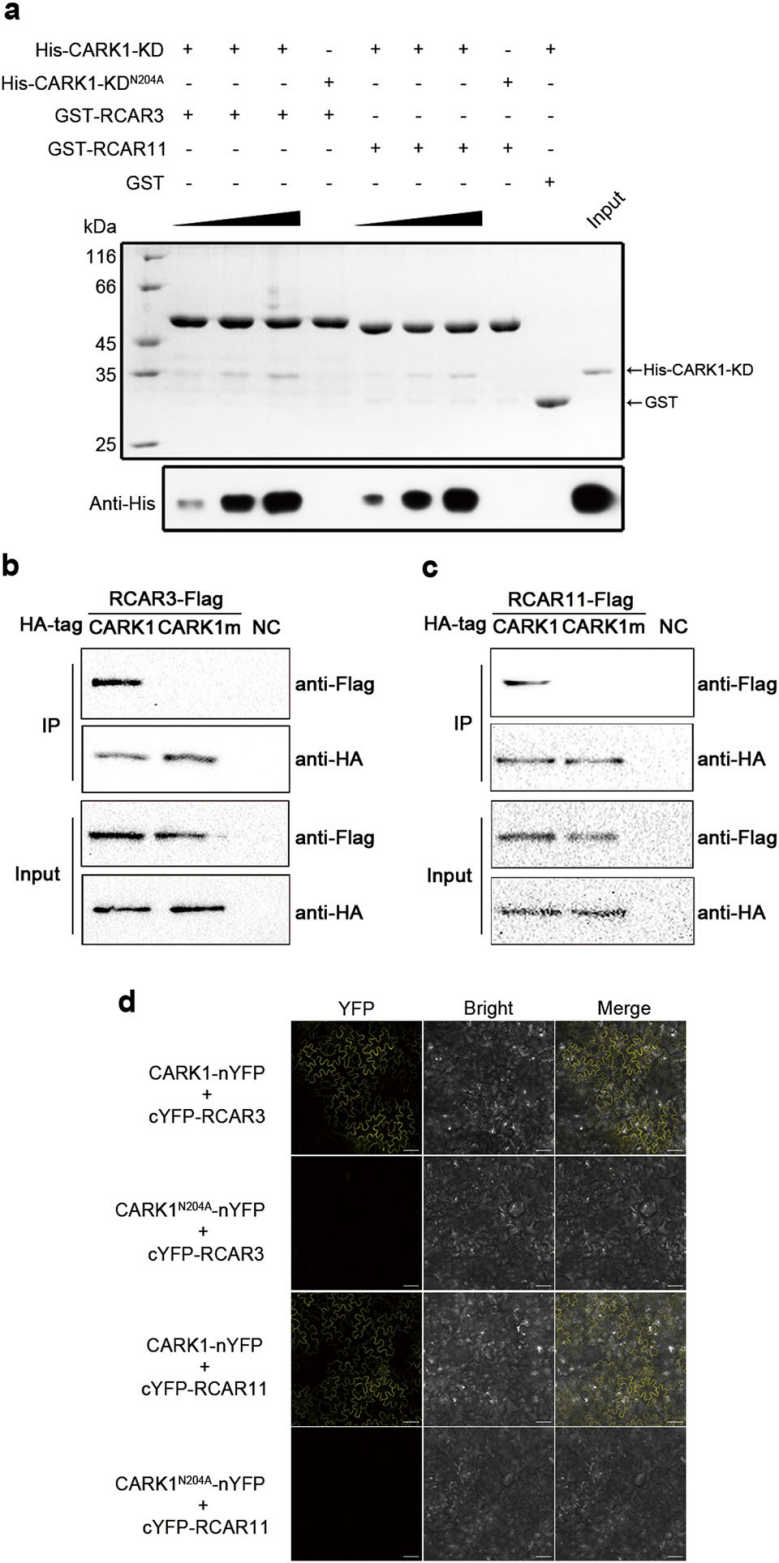
CARK1 interacts with RCAR3 or RCAR11 in vitro and in vivo. **a** GST pull-down assay of CARK1-KD and CARK1-KD^N204A^ interaction with RCAR3 or RCAR11. His-tagged CARK1-KD or CARK1-KD^N204A^ (the amounts with a linear increase from 50 μg, 100 μg and 200 μg) was incubated with 100 μg of GST-tagged RCAR3 or RCAR11, GST, respectively. The samples were analyzed by SDS-PAGE (top) and immunoblot (bottom), the relative protein bands were labeled by the arrow. A quantity of 0.2 μg of input was used. **b**, **c** Co-IP experiment of HA-tagged CARK1/CARK1^N204A^ and Flag-tagged RCAR3 (**b**) or Flag-tagged RCAR11 (**c**). Immunoprecipitates against anti-HA antibody (IP) or crude extracts (Input) were analyzed against anti-HA antibody and subjected to immunoblot analysis against anti-Flag and anti-HA antibodies. Crude extracts without plasmids transfection were used as the negative control (NC). **d** BiFC assay with different combinations of nYFP-CARK1/CARK1^N204A^ and cYFP-RCAR3/RCAR11 in the leaves of *N*. *benthamiana*. Pictures were taken using a confocal laser scanning microscope (LSM 710, Carl Zeiss). Scale bar = 100 µm

The in vitro pull-down assay demonstrated that CARK1-KD interacted with RCAR3 or RCAR11, while these interactions were completely impaired by a single-amino acid substitution in CARK1 (CARK1-KD^N204A^) (Fig. 2a). Co-immunoprecipitation (Co-IP) assay elucidated that the full-length CARK1, but not CARK1^N204A^, could interact with RCAR3 (Fig. 2b) or RCAR11 (Fig. 2c) in Arabidopsis protoplasts. BiFC assay showed that co-expression of CARK1-nYFP/cYFP-RCAR3 or CARK1-nYFP/cYFP-RCAR11 in the leaves of *Nicotiana benthamiana* yielded YFP signals in the cytosol (Fig. 2d), while co-expression of a series of negative controls did not generate fluorescence (Supplementary Fig. S4B). In contrast, co-expression of CARK1^N204A^-nYFP/cYFP-RCAR3 or CARK1^N204A^-nYFP/cYFP-RCAR11 did not yield any YFP signal (Fig. 2d). Our investigations confirm that CARK1 physically interacts with RCAR3 or RCAR11 and the N204 of CARK1 is critical for its interactions with RCAR3 and RCAR11.

### CARK1 phosphorylates RCAR3 or RCAR11 and critically depends on its kinase activity in vitro

**Fig. 3:**
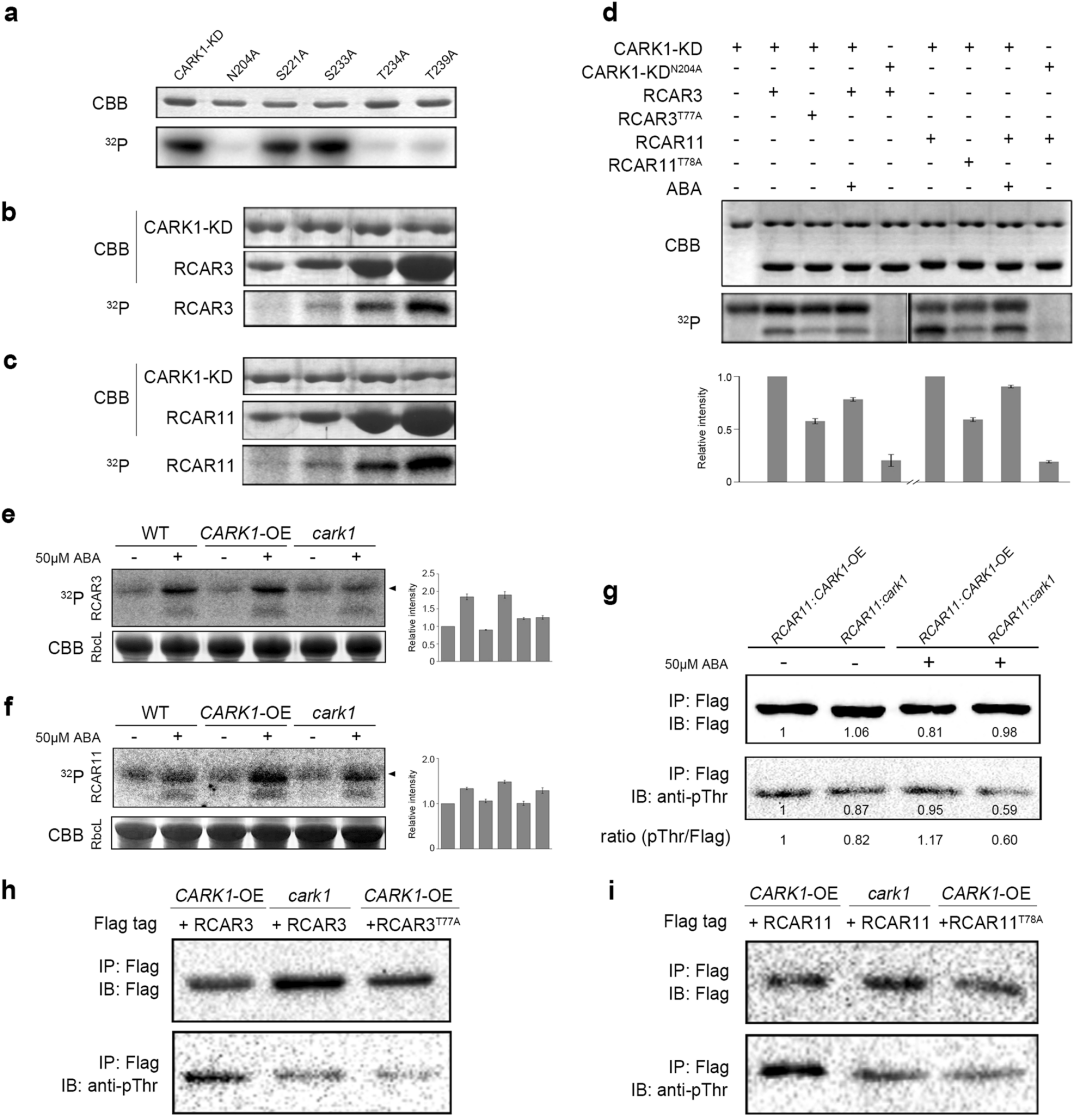
RCAR3 and RCAR11 are substrates of CARK1. **a** Effects of CARK1-KD mutants on its autophosphorylation activity. **b**, **c** Phosphorylation of RCAR3 (**b**) and RCAR11 (**c**) by CARK-KD in vitro. Kinase assay was described in the Methods section. **d** Functional determination of the pivotal phosphorylation site of RCAR3/RCAR11 in vitro. Bottom panel: ^32^P represents autoradiograms, relative radioactivities of RCAR3/RCAR11 was displayed below **d**. Top panel: Coomassie Brilliant Blue (CBB) stained gel. **e**, **f** In-gel kinase assay of WT, *CARK1*-OE and *cark1*. Ten-day-old Arabidopsis seedlings were treated with 50 μM ABA or ddH_2_O (mock) for 1 h. His-tagged RCAR3 (**e**) or His-tagged RCAR11 (**f**) was used as the substrate. In **e**,** f** the arrowheads indicate the ABA-induced bands overlapping with the activated CARK1 (~40 kDa). Right graphs in **e**,** f** show relative radioactivity intensity of ABA-inducible bands (means ± SD, *n* = 3). The relative intensity of non-ABA-inducible bands in WT was normalized to “1”. CBB staining of the large subunit of Rubisco (RbcL) was used as the loading control. **g** Phosphorylation status of endogenous RCAR11 in transgenic plants with or without 50 μM ABA treatment for 1 h. Flag-tagged RCAR11 was immunoprecipitated from the homozygous transgenic plants of *RCAR11*:*CARK1*-OE (#5) and *RCAR11*:*cark1* (#1) and detected by immunoblots. Relative intensities were presented as numbers below each band and the ratio of anti-phosphor-Thr/anti-Flag (pThr/Flag) as indicated. **h**, **i** Phosphorylation of RCAR3/RCAR11 by CARK1 in vivo. Flag-tagged RCAR3/RCAR3^T77A^ (**h**) or RCAR11/RCAR11^T78A^ (**i**) was transfected into the protoplasts of *CARK1*-OE (HA-tag) or *cark1* lines, respectively. Flag-tagged RCAR3/RCAR11 was immunoprecipitated and detected by immunoblots. All experiments were conducted by triplicates with similar results

The results of in vitro kinase assay indicated that wild-type CARK1-KD showed strong autophosphorylation activity (Fig. 3a). However, the kinase activity was abrogated by the active site mutation (CARK1-KD^N204A^) (Fig. 3a). Studies have revealed phosphorylation of residues in the activation loop stabilizes the kinase in an active conformation^25^, so the potential autophosphorylation sites (S221/S233/T234/T239 in CARK1) embedded in the activation loop were analyzed. S221A or S233A substitutions in CARK1 had no observable effect on autophosphorylation, while T234A or T239A substitution resulted in distinct reduction of CARK1 kinase activity, similar to CARK1-KD^N204A^ (Fig. 3a). In the presence of a fixed concentration of CARK1-KD (1.8 μM), phosphorylation of RCAR3 or RCAR11 was positively correlated with their concentrations (Fig. 3b, c). RCAR3 and RCAR11 were phosphorylated by CARK1-KD, but not by CARK1-KD^N204A^ (Fig. 3b, d) and an unrelated kinase from human, Atg1 (Autophagy-related protein 1) and CARK1-KD could not phosphorylate GST, BSA, and the generic substrate MBP^20,21^ (Supplementary Fig. S5A). Point mutation mutants CARK1-KD^T234A^ and CARK1-KD^T239A^ nearly lost their transphosphorylation ability to RCAR3 and RCAR11 (Supplementary Fig. S5B). Hence, the activity of autophosphorylation sites in the activation loop of CARK1-KD is required for phosphorylation of RCAR3 or RCAR11. Besides, phosphorylation of RCAR3 and RCAR11 by CARK1-KD were reduced to some extent in the presence of ABA in vitro (Fig. 3d).

### CARK1 phosphorylates RCAR3/RCAR11 on T77/T78 to promote its stability and inhibitory ability to ABI1 in vitro

Mass spectrometry analysis identified one phosphorylation site on RCAR3, T77 (Supplementary Fig. S6A). Hence, we generated mutant RCAR3^T77A^ and RCAR11^T78A^ (Supplementary Fig. S6B-D) by site-directed mutagenesis. The phosphorylation reactions of RCAR3 and RCAR11 were dramatically decreased, but not abolished in the mutants RCAR3^T77A^ and RCAR11^T78A^ (Fig. 3d), indicating that other phosphorylation sites might exist in the ABA receptors. Nevertheless, T77 of RCAR3 and T78 of RCAR11 do make a major contribution to the phosphorylation by CARK1. By checking the complex structure of PYR1 (RCAR11)-HAB1 (PDB accession code, 3QN1)^26^, T78 is located at the end of the loop between β2 and β3 stand (close to the ‘gate’ loop) in the opposite side of PYR1-HAB1 interface (Supplementary Fig. S6B and C), we explored the possibility that phosphorylation on T77/T78 affects the receptor’s overall folding and stability. The differential scanning fluorimetry (DSF) assay showed an observable increase in thermal stability of phosphor-mimic form of RCAR3 (RCAR3^T77D^) than wild type (melting temperature, Tm, shifts from 36 to 38 °C), consistent with that of CARK1-KD pretreated RCAR3 (Supplementary Fig. S7F). Surface plasmon resonance (SPR) assay further demonstrated the phosphorylation of RCAR3 by CARK1 could distinctly enhance its interaction with ABI1 in vitro. RCAR3^T77D^ binds to ABI1 with a dissociation constant (*K*_d_) of about 0.2 μM, showing a stronger binding affinity than that of wild-type RCAR3 (0.88 μM). By contrast, RCAR3^T77A^ exhibits a much lower binding affinity to ABI1 of about 6.4 μM, similar with the interface point mutant RCAR3^S85R^ (11.3 μM) (Supplementary Fig. S7A-E). Hence, we propose that phosphorylation of T77 on RCAR3 by CARK1 could stabilize the receptor itself and enhance the interaction with Clade A PP2C.

### CARK1 phosphorylates RCAR3 and RCAR11 in planta

To ascertain the phosphorylation activity of CARK1 in vivo, we isolated *CARK1* mutant (*cark1*) and generated lines with ectopic expression of *CARK1* (*CARK1*-OEs) (Supplementary Fig. S8A-C). In-gel kinase assay demonstrated that endogenous CARK1 exhibited its kinase activity by phosphorylating RCAR3 (Fig. 3e) and RCAR11 (Fig. 3f), especially after ABA treatment, the transphosphorylation ability of CARK1 in the *CARK1* overexpression lines (*CARK1*-OE) was significantly activated, but that from the mutant *cark1* showed much reduced kinase activity (Fig. 3e, f). Similarly, the phosphorylation of overexpressed RCAR11 in the *CARK1*-OE line (*RCAR11:CARK1-*OE) was stronger than that in the *cark1* mutant (*RCAR11:cark1*) and also activated by ABA (Fig. 3g; Supplementary Fig. S9). In addition, the phosphorylation status of RCAR3 and RCAR11 was reduced to a great extent when expressing phosphor mutant (RCAR3^T77A^ or RCAR11^T78A^) (Fig. 3h, i). Together these results indicate that RCAR3 and RCAR11 are substrates of CARK1, which may fine-tune ABA signaling by affecting phosphorylation status of ABA receptors.

### CARK1 positively regulates ABA-mediated physiological responses

**Fig. 4:**
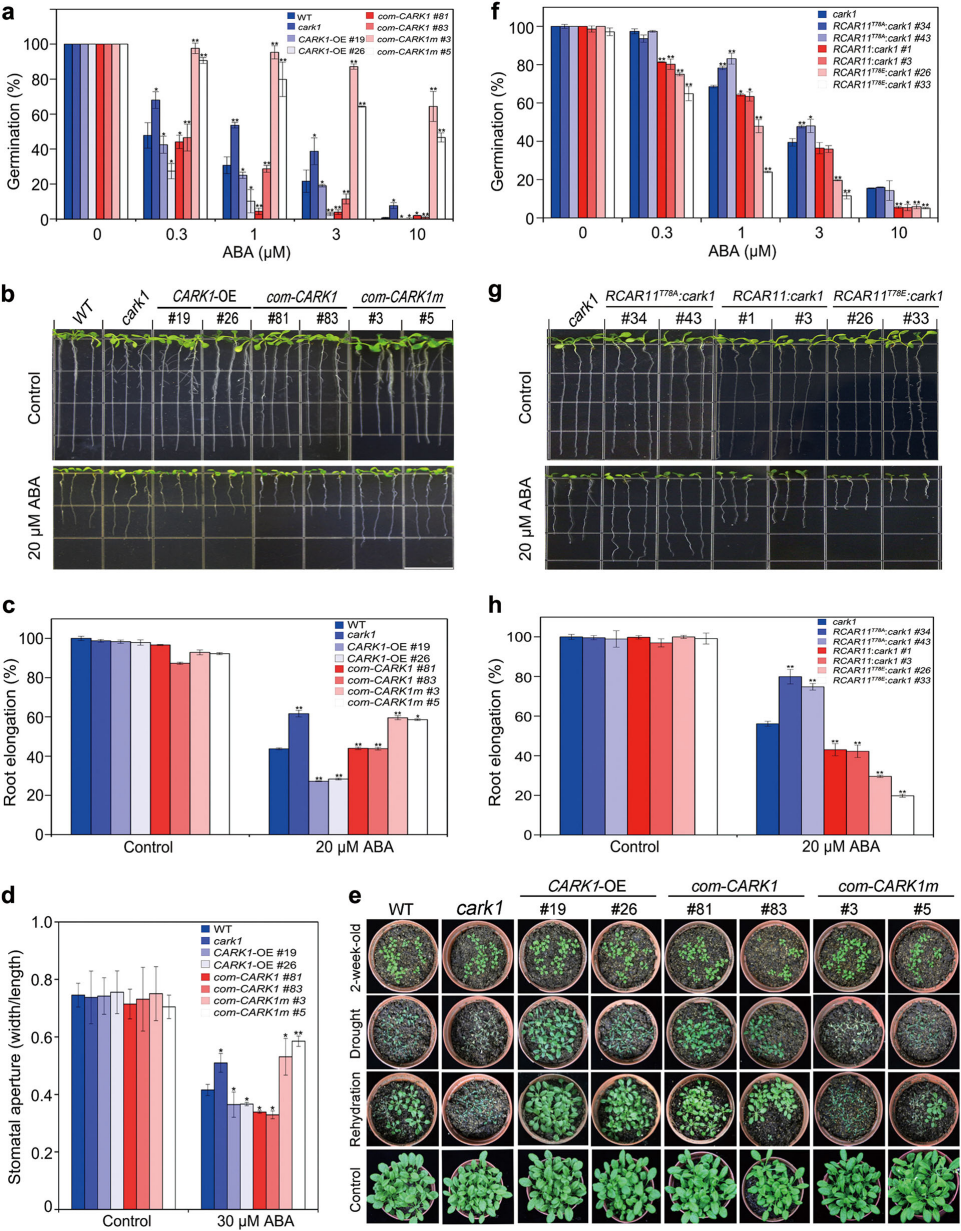
CARK1 positively regulates ABA-mediated physiological responses. **a** Seed germination rates of wild-type Arabidopsis, *cark1* and transgenic plants in the presence of ABA. Seeds (>100) were scored 3 d after stratification on MS medium supplemented with different concentrations of ABA. Values are means ± SD (*n* = 3). **b** The root architecture of wild type, *cark1* and CARK1 transgenic plants seedlings was documented at 7 d post transfer to media (1/2 MS, 1% sucrose) with or without ABA. Scale bar is equivalent to 1.3 cm. **c** Statistical analysis of ABA-inhibited root growth in **b**. Root length is relative to the control (without ABA). Data are means ± SD (*n* = 20). **d** Analysis of ABA-inhibited stomatal opening. Stomatal apertures were measured on epidermal peels of the indicated plants. Data are means ± SD (*n* > 60). **e** Drought tolerance assay. Two-week-old Arabidopsis plants were subjected to drought stress by withholding water for 13 days followed by rehydration for 2 days. Representative images were taken with or without drought stress and after 2 days of rehydration. Control line represents the plants under well-watered conditions. **f** Seed germination rates of *cark1*, *RCAR11*^*T78A*^:*cark1*, *RCAR11*:*cark1*, and *RCAR11*^*T78E*^:*cark1* in the presence of ABA. Seeds (>100) were scored 4 d after stratification on MS medium supplemented with different concentrations of ABA. Values are means ± SD (*n* = 3). **g** The root architecture of *cark1*, *RCAR11*^*T78A*^:*cark1*, *RCAR11*:*cark1*, and *RCAR11*^*T78E*^:*cark1* transgenic plants was documented at 7 d post transfer to media (1/2 MS, 1% sucrose) with or without ABA. Scale bar is equivalent to 1.3 cm. **h** Statistical analysis of ABA-inhibited root growth in **g**. Data are means ± SD (*n* = 20). *P < 0.05, **P < 0.01, Student’s *t*-test. All physiological analyses were conducted by triplicates

*CARK1* is expressed in dry seeds and guard cells (Supplementary Fig. S10)^27^, suggesting that CARK1 is likely involved in ABA-mediated physiological responses. To unambiguously confirm CARK1’s function in ABA signaling, besides *CARK1*-OE plants and *cark1* mutant, we also generated *CARK1* and *CARK1*^*N204A*^ complementation lines in the *cark1* mutant (*com-CARK1* and *com-CARK1m*) (Supplementary Fig. S8C). Seed germination assay demonstrated that *CARK1*-OE and *com-CARK1* plants showed higher sensitivity to ABA than *cark1* mutant and *com-CARK1m* plants (Fig. 4a). Intriguingly, we noticed that *com-CARK1m* had much higher germination rate than *cark1* plants, indicating the dominant-negative effects probably caused by the competition for RCAR targets between CARK1^N204A^ protein (inactive form) and functionally redundant CARK family members. Root growth of *CARK1*-OE and *com-CARK1* plants was more inhibited than those in WT, *cark1* mutant and *com-CARK1m* plants in the presence of ABA (Fig. 4b, c). Analysis of stomatal apertures in response to ABA indicated *cark1* mutant and *com-CARK1m* impaired ABA inhibition of stomatal opening, while stomata of *CARK1*-OE and *com-CARK1* plants were more inhibited than WT (Fig. 4d). During dehydration period, WT, *cark1* mutant and *com-CARK1m* plants wilted, while *CARK1-*OE, *com-CARK1* did not show wilted phenotypes (Fig. 4e). After rehydration, *CARK1*-OE and *com-CARK1* plants recovered much better than other plants (Fig. 4e). The transcript abundance of ABA-inducible genes, such as *RAB18, RD29A* and *RD29B* was enhanced in *CARK1-*OE plants compared to WT, but reduced in the *cark1* mutant plants (Supplementary Fig. S11A-C). Therefore, CARK1 positively regulates ABA-mediated physiological responses. Subsequently, we generated various *RCAR11* transgenic lines in *cark1* mutant, using wild type, phosphor-defective and phosphor-mimic forms on T78 of RCAR11 (Supplementary Fig. S8D). Seed germination and root elongation data consistently showed that transgenic plants bearing phosphor-defective form of RCAR11 (*RCAR11*^*T78A*^:*cark1*) nearly lost ABA sensitivity compared to wild-type form of RCAR11 (*RCAR11*:*cark1*), while phosphor-mimic form of RCAR11 (*RCAR11*^*T78E*^:*cark1*) could notably recover ABA sensitivity (Fig. 4f, g). Taken together, these results further reveal the significance of phosphorylation of ABA receptors by CARK1 in fine-tuning ABA signaling.

### CARK1 enhances the expressions of ABA-responsive genes and the inhibition of RCARs/PYR/PYLs on ABI1

Transfection of protoplasts with CARK1 enhanced the expression of *RAB18-LUC* and *RD29B-LUC* in the absence or presence of ABA (Supplementary Fig. S11D and E), which is consistent with the results of qRT-PCR analyses. However, transfection of protoplasts with CARK1^N204A^ reduced this effect (Supplementary Fig. S11D and E). To test the effect of phosphor-mimic form of RCAR3/RCAR11 (RCAR3^T77D^/RCAR11^T78D^) on ABI1 activity, in vitro enzymatic assay was employed. In the absence of ABA but wild-type RCAR3, the phosphatase activity of ABI1 was reduced by ~22% compared with that of ABI1 alone, while wild-type RCAR11 could hardly block ABI1 activity, consistent with other groups’ published data^5,6^. Notably, this inhibitory effect caused by RCAR3^T77D^ and RCAR11^T78D^ could be significantly magnified to the level of ~42 and 28% than wild-type RCAR3/RCAR11 without ABA, respectively (Supplementary Fig. S11F). In the presence of ABA, the phosphatase activity of ABI1 was totally abolished by either forms of RCAR3/RCAR11 (Supplementary Fig. S11F). Together, we propose that CARK1 enhances the ability of RCAR3/RCAR11 to inhibit ABI1 activity via phosphorylation at the conserved threonine residue. Hence, these studies further support that kinase activity is critical for the function of CARK1 in ABA signaling.

## Discussion

Here we report that CARK1 is identified as the kinase responsible for RCAR3 and RCAR11 phosphorylation and plays a crucial role in the positive regulation of ABA signal transduction in Arabidopsis. Actually, to fully understand the relationship between CARK1 and RCARs, we used CARK1 as the prey in yeast assay to examine its interplay with all 14 members of ABA receptors which act as the bait individually. As shown in the result (Supplementary Fig. S1B), CARK1 interacts with all the RCAR family members except RCAR7 (PYL13) and RCAR14 (PYL2). Considering the monomeric/dimeric states of ABA receptor, RCAR3 (monomer) and RCAR11 (dimer) were finally chose as the representative in this study. Additionally, CARK1 also belongs to a multi-gene family, we cannot exclude the possibility that other RLCKs may also interact with RCARs. Previous studies had indicated that proteins with high-sequence similarities to CARK1 in *Solanum lycopersicum* (Pti1) and Arabidopsis (Pti1-1/2/3/4 and MRI) were involved in disease resistance^28^, oxidative stress response^20,21^ and pollen tube growth^22,23^, however evidence of their involvement in ABA related response was little mentioned. Indeed, although CARK1 family seems function diversely in biotic and abiotic stresses, it may display partially functional redundancy that matters to the evolution, which has been implicated in our biochemical data. Meanwhile, with the myristoylation site at the N-terminal, CARK1 may be involved in the membrane-associated signaling cascades under certain stress, like the action mode of BIK1, a RLCK in plant innate immunity^29^, which could facilitate to characterize the upstream kinase of CARK1.

Most recently, Wang et al.^30^. reported the Target of Rapamycin (TOR) kinase could phosphorylate PYL ABA receptors at a conserved serine residue of β4 strand, which facilitates plants to utilize the phospho-regulatory mechanism for the balance of growth and stress responses. Although same as the ABA receptor phosphorylation, unlike the positive regulation caused by CARK1 on a conserved threonine/serine residue of the loop between β2–β3 strand, TOR kinase negatively modulates ABA signaling by phosphorylating PYL ABA receptors. Therefore, different phosphorylation sites by kinases could scale-up for diverse regulatory effects, which may account for the versatile function of plants in response to stresses.

Considering that the inconsistency observed in vitro (Fig. 3d) and in vivo data (Fig. 3e–g) about the effect of ABA on the receptor (RCAR3 and RCAR11) phosphorylation by CARK1, we would like to clarify and propose that the relatively unitary reaction system in vitro could not truly mimic the complicated environment in vivo. Based on that, we concluded that the activity of endogenous CARK1 on RCAR3/RCAR11 was highly motivated by ABA, which was confirmed through the serials of experimental data in planta afterwards (Fig. 3e–g).

The physiological and biochemical analyses have corroborated the potential regulatory mechanism of CARK1 in the manner of directly phosphorylating RCARs in ABA signaling. Due to the ambiguous bias of residue serine and threonine for a Ser/Thr protein kinase, it is rational to speculate receptors with Ser corresponding to Thr77 of RCAR3 or Thr78 of RCAR11 can also be phosphorylated by CARK1. The activity of RCARs, which is modulated by phosphorylation, evinces a novel fine-tuning mechanism in ABA signaling. Characterization of CARK1 expands our understanding of the regulatory aspects of ABA transduction network beyond previous finding of the core components of ABA signaling^26^. In conclusion, CARK1 acts as a novel positive regulator of ABA signaling by phosphorylating ABA receptors.

## Materials and methods

### Plant materials and growth conditions

Arabidopsis plants used in this study were all in the Columbia (Col-0) background. The *CARK1* T-DNA insertion mutant *cark1* (SALK_113377) was obtained from the Arabidopsis Biological Resources Center. Plant was grown on soil-vermiculite mixtures at 22 °C under 60% relative humidity with cycles of 16 h light and 8 h dark. For plate culture, seeds were first soaked in distilled water for 3 days at 4 °C. After stratification, seeds were surface sterilized and germinated on solid MS medium containing 2% sucrose and 0.8% agar, pH 5.9.

### Yeast two-hybrid analysis

Arabidopsis ds cDNAs were amplified using RT-PCR followed by LD-PCR and purification, then cloned into *Sma* I-linearized pGADT7-Rec vector (Clontech, CA, USA). A cDNA of *RCAR3* was cloned into the pGBKT7 vector (Clontech, CA, USA) digested with *Eco* RI and *Pst* I. Yeast two-hybrid analyses were performed using the MatchMaker GAL4 Two-Hybrid System 3 (Clontech, CA, USA) according to the manufacturer’s instructions. Sequence analyses revealed that two positive clones containing amino acid residues 290–364 of CARK1 (hereafter referred to as CARK1C) showed a strong interaction with RCAR3. To verify the interaction between the CARK1C and RCARs (all 14 receptor members), CARK1C was cloned into pGADT7 via *Eco* RI and *Xho* I sites, and RCARs was cloned into pGBKT7 via *Eco* RI and *Bam* HI sites. To test the effect of ABA on the interaction between CARK1C and RCARs, 10 μM ABA was added into the indicated medium. Primers used in yeast two-hybrid assay are listed in Supplementary Table 1.

### X-ray crystallography

For protein preparation, wild-type CARK1-KD (residues 50–353) was expressed in *Escherichia coli Rosetta* (DE3) as 6× His fusion protein and purified following the general method as described in Supplementary Information. Proteins used in this study were purified with the similar procedures as described for CARK1-KD. For details of crystallization, data collection and structure determination, see Supplementary Information. Data collection and refinement statistics are summarized in Supplementary Table 2.

### In vitro pull-down assay

The cDNA of RCAR3/RCAR11 was cloned into pGEX-6P-1 vector to produce GST fusion proteins, and the cDNA fragment of CARK1-KD/CARK1-KD^N204A^ was cloned into pET28a vector to produce His fusion proteins. For more details of the assay, see Supplementary Information.

### Bimolecular fluorescence complementation (BiFC) assays

The cDNAs of *CARK1*, *CARK1*^N204A^, *RCAR3*, and *RCAR11* were subcloned into the binary nYFP or cYFP vector via *Kpn*I/*Mlu*I or *Kpn*I/*Sal*I. For more details, see Supplementary Information. Primer pairs for the construction of vectors are listed in Supplementary Table 1.

### Co-immunoprecipitation (Co-IP) assay

Different combinations of HA-tagged CARK1/CARK1^N204A^ and Flag-tagged RCAR3/RCAR11 were co-transfected into wild-type Arabidopsis protoplasts, after extraction in IP buffer, crude protein extracts (Input) were used for immunoprecipitation with Anti-HA Magnetic Beads (Pierce, Illinois, USA). IP buffer containing 50 mM Tris, pH 7.5, 150 mM NaCl, 5 mM EDTA, 1% Triton X-100, 5 mM NaF, 1 mM DTT, 1× complete protease inhibitor cocktail (Roche, Basel, Switzerland) was used for lysis and wash. After wash, the beads were resuspended in 5× sample loading buffer and boiled at 100 °C for 5 min. Supernatant obtained from the crude extracts was used as the input. HRP-conjugated anti-HA and HRP-conjugated anti-Flag antibodies (Bioworld, Minneapolis, USA) were used in the immunoblot. Crude extracts with no exogenous plasmids were used as the negative control (NC). For other immunoprecipitaton experiments, see details in Supplemental Information.

### In vitro kinase assay and PP2C phosphatase assay

The in vitro kinase assay was performed as described^31^. The phosphatase activity was measured by the serine/threonine phosphatase assay system (Promega, WI, USA). Details are provided in Supplemental Information.

### In-gel kinase assay

The in-gel kinase assay was performed as described^32^ with some modifications. Total proteins were extracted from 10-d-old seedlings after treatment with 50 μM ABA or ddH_2_O (mock) for 1 h. The extraction buffer contains 50 mM HEPES, pH 7.5, 5 mM EDTA, 5 mM EGTA, 1 mM Na_3_VO_4_, 25 mM NaF, 20% glycerol, 2 mM DTT, 1× protease inhibitor cocktail (Roche, Basel, Switzerland). Equal amounts of total protein (150 μg) were loaded on 10% SDS-PAGE gel embedded with 0.8 mg of His-RCAR3. A total of 80 μCi of [γ-^32^P]-ATP was used for one gel and the kinase reaction was performed at RT for 3 h. Dried gel was exposed with phosphor screen followed by Typhoon 9410 imager and the signal intensity was quantified by ImageJ.

### Genotyping analysis of *CARK1* mutant

To isolate the homozygous *cark1* mutant, genomic DNAs were extracted from rosette leaves and subjected to PCR analysis using gene-specific primers FP and RP and T-DNA-specific primer LBb1.3. PCR products were separated by agarose gel electrophoresis and visualized with ethidium bromide (EB) staining. Homozygous mutant was propagated and used for the subsequent experiments. Primers used in this assay are listed in Supplementary Table 1. For the generation of other transgenic plants, see details in Supplemental Information.

### Physiological analysis

For germination assay, seeds grown under the same conditions were collected from mature siliques of various plants on the same day and were air dried at 30 °C for about 2 weeks. Then, more than 100 seeds from each line were placed on solid MS medium containing different concentrations of ABA.

For root growth assay, >100 seeds from each line were first germinated vertically on 1/2 MS medium for 3 days. Then, 20 seedlings of each line with similar root length were transferred to 1/2 MS medium supplemented with or without 20 μM ABA in vertical position. The root length was determined 1 week after transfer.

For stomatal aperture measurement, 4-week-old seedlings were cultured in darkness for 24 h to make the stomata close. Then, epidermal strips were peeled and immersed under light in opening solution containing 10 mM MES-KOH (pH 6.15), 10 mM KCl and 50 μM CaCl_2_ with or without 30 μM ABA for 3 h at 22 °C. Stomatal apertures were examined and photographed with a fluorescence microscope (DMI6000B, Leica). Width/length ratios of >60 stomata from each line were measured with Leica Application Suite software.

For drought tolerance test, 2-week-old seedlings from each line grown under the same conditions were firstly subjected to drought stress treatment by withholding water for 13 days. Then, 2 days after rehydration, the morphological changes of plants were recorded.

### Statistical analysis

The data are represented as means ± SD. Statistical analysis was performed using Student’s *t*-test, with the use of SPSS Software, Version 16.0. Values of *P* < 0.05 were considered significant, and Values of *P* < 0.01 were considered more significant.

### Accession code

The atomic coordinates and structure factors for the reported crystal structure have been deposited in the Protein Data Bank (PDB) under the accession code 5XD6.

## Electronic supplementary material

SUPPLEMENTAL MATERIAL
