# Supplementary material for: CARK1 mediates ABA signaling by phosphorylation of ABA receptors
Source: Cell Discov. 2018 Jun 19;4:30. doi: 10.1038/s41421-018-0029-y (PMC6006248; doi:10.1038/s41421-018-0029-y)
Supplement: Supplementary file 1 — SUPPLEMENTAL MATERIAL [file 41421_2018_29_MOESM1_ESM.pdf]

Supplementary Information for:

CARK1 mediates ABA signaling by phosphorylation of ABA receptors

Methods

Protein preparation

CARK1-KD (residues 50-353) was cloned into the pET28a expression vector (Novagen, WI, USA) with a 6 × His tag fused at the N-terminal side. The plasmid was then transformed into *E. coli* strain *Rosetta* (DE3), and the transformed cells were cultured at 37℃ in LB media containing 50 mg/L Kanamycin. After OD_{600} reached 0.5, the culture was cooled to 16℃ and supplemented with 0.5 mM IPTG. After overnight induction, the cells were harvested by centrifugation and the pellets were resuspended in lysis buffer containing 20 mM Tris-HCl (pH 8.0), 150 mM NaCl and 5 mM $MgCl_2$ followed by homogenization using an ultra-high-pressure cell disrupter (JNBIO, Guangzhou, China) at 4℃. The insoluble material was removed by centrifugation at 20,000 g. The fusion protein was first purified by Ni-NTA (Qiagen, Hilden, Germany) affinity chromatography and eluted with wash buffer containing 30 mM imidazole in the lysis buffer. The bound target protein was subsequently eluted with a buffer containing 300 mM imidazole in the lysis buffer. The eluted protein was further purified by a HiTrap Heparin HP column (GE Healthcare, PA, USA) and Superdex-75 gel filtration column (GE Healthcare, PA, USA). The purified CARK1-KD was > 95% pure according to SDS-PAGE and then was concentrated to 50 mg/mL in 20 mM Tris-HCl (pH 8.0), 100 mM NaCl and 5 mM $MgCl_2$ and stored at -80℃. Glycerol at a final concentration of 10% (v/v) was added into protein stocks used for *in vitro* kinase assay.

RCAR3 and RCAR11 were cloned into the pET28a expression vector with 6 × His tag fused at the C-terminal side and the pGEX-6p-1 expression vector (Novagen, WI, USA) with GST tag fused at the N-terminal end. These plasmids were transformed into *E. coli* strain *Rosetta* (DE3), protein expression and purification were performed as

described for CARK1-KD and the purified proteins were stocked in 20 mM Tris-HCl (pH 8.0), 100 mM NaCl, 1 mM DTT except that the proteins used in the SPR assay were stocked with 20 mM HEPES, pH 7.4, 150 mM NaCl. Mutants of CARK1-KD and RCAR3 or RCAR11 were obtained by site-directed mutagenesis using *Fast* Mutagenesis System (TransGen Biotech, Beijing, China) and further purified using the same procedures as for wild-type protein.

ABI1 (residues 117-434) was cloned into pGEX-6p-1 expression vector and transformed into *E. coli* strain *Rosetta* (DE3). Protein expression was performed as described for CARK1-KD. The proteins were purified with glutathione S-Sepharose 4B resin (GS4B, GE Healthcare, PA, USA) in the buffer containing 50 mM HEPES (pH 7.5), 200 mM NaCl, 5 mM $MgCl_2$ and 5% glycerol, and the GST tag was removed by PPase digestion at 4℃ for overnight before size-exclusion chromatography (Superdex-200, GE Healthcare) and the purified protein was stocked in 50 mM HEPES (pH 7.5), 100 mM NaCl, 5 mM $MgCl_2$ and 5% glycerol.

Crystallization and Structure Determination

Crystallization was performed at 18℃ by the hanging-drop vapor-diffusion technique. Crystals were obtained by mixing 1 μL of protein solution (35mg/mL) with an equal volume of reservoir solution, and then equilibrated against 200 μL of reservoir solution. The crystals for native CARK1-KD in complex with AMP-PNP were obtained in a reservoir solution containing 0.1 M ammonium acetate, 0.1 M Bis-Tris (pH 5.5), and 17% (w/v) PEG 10,000, supplemented by 3 mM AMP-PNP. Plate-like rhombic crystals appeared overnight and typically grew to full size within 5 days. The selenomethionine derivatives of CARK1-KD were purified following a general procedure, and then crystallized under similar conditions to the native protein. All crystals were gradually transferred into a harvesting solution containing the respective precipitant solutions plus 10% (v/v) glycerol before being flash frozen in liquid nitrogen for storage. Data were collected under cryogenic conditions at 100 K. The selenomethionine SAD data set of the AMP-PNP-bound CARK1-KD at the Se peak wavelength was collected at the Photon Factory (PF,

Tsukuba, Japan) beamline BL17A at 2.4 Å, and another native data set was collected in Shanghai Synchrotron Radiation Facility (SSRF) beamline BL17U at 1.9 Å. All data sets were processed using the HKL2000 package. The SAD data phases were calculated and substantially improved by solvent flattening using the PHENIX program[1]. A model was manually built into the modified experimental electron density by using COOT[2] and further refined in PHENIX. The Se-Met refined model was used for molecular replacement to phase the native protein. Model geometry was verified using the program MolProbity. The final refinement statistics are summarized in Supplementary Table 2. Structural figures were drawn using the program PyMOL.

***In vitro* pull-down assay**

100 μg of GST-RCAR3 were added to 25 μL of glutathione S-Sepharose 4B (GS4B, GE Healthcare, PA, USA) resin. 100 μg of catalytic-defective mutant CARK1-KD^{N204A} and different amount of His-tagged wild-type CARK1-KD (50 μg, 100 μg and 200 μg) were incubated with the RCAR3-bound resin for 1 h at room temperature, respectively. The resin was then extensively rinsed with buffer to remove unbound proteins. The resin was finally resuspended with 100 μL of buffer and 10 μL of suspension were applied to 12% SDS-PAGE for analysis. Pull-down assays for RCAR11 were conducted in the same way as described for RCAR3. GST was considered as a negative control, 0.2 μg of inputs was used in the assay. The anti-His antibody (mouse derived Mab, TransGen Biotech; Beijing, China) was used in the Western blot analysis.

Subcellular localization assay and Bimolecular Fluorescence Complementation (BiFC) assay

For subcellular localization assay, the ORFs of *CARK1* were cloned in to the pBI221 vector containing the CaMV 35S promoter, resulting in the pBI221-CARK1-GFP plasmid. For the transient expression of CARK1-GFP in Arabidopsis mesophyll protoplast, the corresponding pBI221-CARK1-GFP plasmid was transformed using the PEG-calcium transfection method as described[3]. After transformation, the mesophyll protoplasts were kept in darkness at room temperature for 16h before examination by confocal fluorescence microscope (Leica, Germany).

For BiFC assay, the full-length coding sequences of *CARK1* and *CARK1*^{N204A} were subcloned into the binary vector nYFP to generate CARK1/CARK1^{N204A}-nYFP plasmids via *Kpn* I and *Mlu* I sites, and the full-length coding sequences of *RCAR3* and *RCAR11* were subcloned into the binary vector cYFP to generate cYFP-RCAR3/RCAR11 plasmids via *Kpn* I and *Sal* I sites. Primer pairs for the construction of vectors are listed in Supplementary Table 1. The assay was performed as described[4]. *Agrobacterium tumefaciens* strain GV2260 with indicated nYFP or cYFP vectors were incubated, harvested, and resuspended in infiltration buffer (0.2 mM acetosyringone, 10 mM $MgCl_2$, and 10 mM MES) to identical concentrations (OD_{600} 0.5). Equal concentrations and volumes of *Agrobacterium* strains were mixed and coinfiltrated into *N. benthamiana* leaves with a needleless syringe. After infiltration, plants were placed at 24 °C for 48 h before observation. YFP fluorescence was detected with a Zeiss microscope (LSM710, Jene, Germany) and analyzed using the ZEN 2009 software. The experiment was repeated three times, each time with 3 biological replicates.

Immunoprecipitation assay

For immunoprecipitation, Flag-tagged RCAR3/RCAR3^{T77A} or Flag-tagged RCAR11/RCAR11^{T78A} was transfected into *CARK1*-OE and *cark1* protoplasts or the transgenic plants of *RCAR11*:*CARK1*-OE and *RCAR11*:*cark1* with Flag-tagged RCAR11 were used for the immunoprecipitation assay. After extraction in IP buffer (50 mM Tris, pH 7.5, 150 mM NaCl, 5 mM EDTA, 1% Triton X-100, 5 mM NaF, 1 mM DTT, 1×Complete protease inhibitor cocktail), crude protein extracts were immunoprecipitated with ANTI-FLAG M2 Magnetic Beads (Sigma, Missouri, USA). For detection of the phosphorylation variations caused by CARK1 and its catalytic-defective mutant CARK1^{N204A} under physiological conditions, Flag-tagged RCAR3/RCAR3^{T77A} or Flag-tagged RCAR11/RCAR11^{T78A} was normalized to approximately equal amount using HRP-conjugated anti-Flag antibody (Bioworld, Minneapolis, USA). HRP-conjugated Phosphor-Threonine Antibody (Cell Signaling Technology, Beverly, MA) was used. Western blots were developed with the ECL chemiluminesence detection system (Bio-Rad,

California, USA). For the phosphorylation variation of endogenous RCAR11 in *RCAR11*:*CARK1*-OE and *RCAR11*:*cark1* transgenic plants, Flag-tagged RCAR11 was immunoprecipitated and detected as above.

Enzymatic analyses

a) In vitro kinase assay

The reaction buffer contained 20 mM Tris (pH 7.5), 100 mM NaCl, 10 mM $MgSO_4$, 2 mM DTT, 2.5 μCi [γ-^{32}P]-ATP (3000 Ci/mmol, PerkinElmer; MA, USA). For autophosphorylation and transphosphorylation assays, 1.8 μM of CARK1-KD and various CARK1-KD mutants were diluted to 25 μL using the reaction buffer. To determine the transphosphorylaiton effect of CARK1-KD on RCAR3 and RCAR11, and to facilitate the choice of appropriate amount of RCAR3 and RCAR11 in the assay, kinase assays were conducted in the presence of different concentrations of RCAR3 and RCAR11 (3.11 μM, 9.33 μM, 28 μM, and 84 μM). To determine the effect of ABA on CARK1-KD activity and to analyze CARK1-KD activity on RCAR3/RCAR11 phosphor mutants (RCAR3^{T77A} and RCAR11^{T78A}), 1.8 μM of CARK1-KD, 28 μM of RCAR3 or RCAR3^{T77A}, and 28 μM of RCAR11 or RCAR11^{T78A} were mixed in 25 μL of reaction buffer, 10 μM of (+)-ABA (Sangon Biotech, Shanghai, China) was used in the assay. The reaction mixture was incubated at 30 °C for 1 h and terminated by adding an equal volume of 2 × SDS loading buffer. All proteins tested were tagged with 6 × His and purified from *E. coli* individually. Protein samples were separated by Tricine polyacrylamide electrophoresis, and phosphorylated proteins were visualized by a phosphoimager.

b) PP2C Phosphatase Assay

Reactions were performed in a 5× reaction buffer containing 250 mM imidazole, pH 7.2, 1 mM EGTA, 25 mM $MgCl_2$, 0.1% β-mercaptoethanol and 0.5 mg/ml BSA, 0.3 μM ABI1 (residues 117-434), 3 μM WT or mutant RCAR3 or RCAR11 proteins and 10 μM (+)-ABA (Sangon Biotech, Shanghai, China) were added if required. After

incubation with peptide substrate (RRA(pT)VA) at 30 °C for 20 min, the reaction was stopped by addition of 50 µl molybdate dye. Absorbance at 630 nm was measured 20 min after the addition of molybdate dye. The values shown were normalized to the control (ABI1) and expressed as % of ABI1 activity.

Sample Preparation for Mass Spectrum Analysis

Protein samples were prepared as described above, and the recombinant RCAR3 and RCAR11 were first incubated with CARK1-KD at a ratio of 10:1 (w/w) in the kinase buffer (20 mM Tris–HCl, pH 7.5, 10 mM $MgCl_2$, 5 mM EGTA, 100 mM NaCl, 1 mM DTT) supplemented with 10 mM ATP at 30 °C for 3 h with gentle shaking. After the reaction, the protein mixture was digested with trypsin (Promega, WI, USA) at a ratio of 1:40 (E:S) overnight. Phosphopeptides present in the tryptic digests were dried in a speed vacuum and reconstituted in 0.1% FA/H_2O for LC-MS/MS analysis.

LC-MS/MS analysis for identification of protein phosphorylation

Peptide samples were first loaded onto a Waters Symmetry C18 trapping column (300 µm i.d. × 1 cm length) at a flow rate of 8 µL/min using Waters NanoAcquity UPLC system. After desalting and pre-concentration, peptides were separated by in-line gradient elution onto a 100 µm i.d. × 10 cm column packed with 1.7 µm BEH C18 material (Waters, MA, USA) at a flow rate of 400 nL/min using a linear gradient from 2% to 35% B over 30 min (A = 0.1% FA in H_2O, B = 0.1% FA in ACN). The Waters Synapt Q-IM-ToF mass spectrometer was operated in high-definition MSE mode (high- and low-collision energy switching every 1.0 s), and the data were processed with ProteinLynx Global Server (PLGS version 2.4; Waters) to reconstruct MS/MS spectra by combining all masses with a similar retention time. MS/MS spectra were searched against a house-made database consisting of RCAR3 and RCAR11 sequences with the following parameters: peak width, 0.3 min; MS and MS/MS tolerance, automatic (usually below 20 ppm); trypsin missed cleavages, 1; fixed modification, carbamidomethylation; variable modifications, Met oxidation and phosphorylation of Ser, Thr, or Tyr; and false positive rate, 4%.

Differential scanning fluorimetry assay

The thermal stabilities of various RCAR3 were determined by differential scanning fluorimetry (DSF) using a real-time CFX96 thermocycler (Bio-Rad, California, USA) instrument. Proteins (5 μM of RCAR3/RCAR3^{T77D}, 0.5 μM of CARK1-KD) were incubated with the dye SYPRO orange (5×) and heated in stepwise increments of 1℃ per min from 25℃ to 95℃. The wavelength for excitation was set to 490 nm and emission was set to 575 nm for SYPRO orange. The melting temperature (Tm) was defined as the temperature at which the fluorescence increase was 50% of the maximal increase.

Surface plasmon resonance analyses

For kinetic experiments, a Biacore T200 instrument (GE Healthcare, PA, USA) was used according to the manufacturer's manual. Purified ABI1 (residues 117-434, 20 μg/ml in 10 mM sodium acetate, pH 5.5) was immobilized onto the CM5 sensor chip (GE Healthcare, PA, USA) at 2645 response unit (RU) using an amine-coupling kit (GE Healthcare, PA, USA). HBS-EP, which contains 10 mM HEPES, pH 7.4, 150 mM NaCl, 3 mM EDTA, and 0.05% (v/v) Tween 20, was used as the running buffer. The sensorgram was collected at 25℃ at a constant flow of 30 μl/min to determine the binding affinity. Serially diluted concentrations (0.259 μM, 0.778 μM, 2.333 μM, 7 μM and 21 μM) of RCAR3 or its variants (RCAR3^{T77D}, RCAR3^{T77A} and RCAR3^{S85R}) were injected through the chip surface for 165 s, followed by a dissociation period for 480 s. 10 mM NaOH was used as the regeneration reagent. Sensorgrams were subjected to global analysis using the 1:1 Langmuir binding model by BIAevaluation software. Accuracy of the model fitting was described by $\chi 2$ parameter. In this assay, RCAR3^{S85R} was chosen because S85 from RCAR3, corresponding to S112 in PYL1 (RCAR12) or S89 in PYL2 (RCAR14)[5], likely to mediate the interaction between RCAR3 and ABI1.

Generation of various CARK1 and RCAR11 transgenic plants

To generate the construct of *CARK1*-OE plants, HA-*CARK1* fragments (containing one copy of HA at the N-

terminus of *CARK1* cDNA) were amplified and cloned into the *Xba* I and *Sac* I sites of the pBI121 vector (Clontech, CA, USA) to replace the β-glucuronidase (GUS) coding sequence. Then, the resulting construct was transformed into *Agrobacterium tumefaciens* strain GV3101, which was subsequently infiltrated into the wild-type plants using the "floral dip" method[6]. To generate the complementation lines, HA-*CARK1* and HA-*CARK1*^{N204} (denoted HA-*CARK1m*) over-expressing plants in *cark1* mutant background were confirmed in 10-day-old seedlings compared to wild-type or transgenic plants by RT-PCR. Transgenic plants over-expressed Flag-tagged RCAR11 in the background of *CARK1*-OE or *cark1* mutant (denoted *RCAR11:CARK1*-OE and *RCAR11:cark1*) and the transgenic lines of Flag-tagged *RCAR11/RCAR11*$^{T78A/E}$ in the *cark1* mutant (denoted *RCAR11*$^{T78A/T78E}$*:cark1*) were generated as previously mentioned. All transgenic plants were screened on MS medium supplemented with kanamycin or hygromycin and mRNA levels were verified with RT-PCR assays.

Protoplast isolation and transient activation assay

The constructs used in this assay are all derivatives of pBI221. *CARK1* and *CARK1*^{N204A} were generated by replacing the glucuronidase (GUS) gene in the vector pBI221 with *haemagglutinin (HA)-CARK1* or *HA-CARK1*^{N204A} fragments via *Xba* I and *Ecl*136 II sites. A dual-luciferase (Dual-LUC) method was used for the transient activation assay as described[7]. A 527bp and 773bp region of the *Arabidopsis* RAB18 and RD29B promoter were amplified by PCR and subcloned into pGreenII 0800-LUC by digestion with *Hind*III and *BamH*I, respectively. The pGreenII 0800-LUC vector encodes two luciferases, the firefly luciferase controlled by the recombinant RAB18/RD29B promoter, and the Renilla luciferase (RLUC) controlled by the constitutive 35S promoter. Primers used in this study are listed in Supplementary Table 1.

Preparation of Arabidopsis protoplasts of the wild-type plants and subsequent transfection of protoplasts were performed as described[3]. The dual-LUC vector containing *pRAB18::FLUC* or *pRD29B::FLUC* was used at 10 μg per transfection, *CARK1* and *CARK1*^{N204} were also used at 10 μg per transfection. In addition, extra empty plasmids

were added to maintain consistency of the total amount of plasmids per transfection. When indicated, 10 μM ABA was added into the incubation buffer immediately after transfection. Luciferase activity was measured by an LMax II^{384} luminometer (Molecular Devices, Bad Wildbad, Germany) using the Dual-Luciferase Reporter Assay System from Promega (Madison, WI, USA). Relative FLUC activity was calculated by normalizing against the RLUC activity, and the data presented were the averages of three biological replicates.

RNA isolation and qRT-PCR analysis

Two-week-old seedlings were incubated in liquid MS medium with or without 10 μM ABA for 2 h. Total RNAs were extracted using the RNAiso Plus reagent from Takara (Otsu, Japan). The cDNA was synthesized from 1 μg of total RNAs using PrimeScript RT-PCR reagent Kit of Takara. qRT-PCR was performed using the Bio-Rad CFX96 real-time PCR detection system (Bio-Rad, California, USA) and SYBR Premix Ex Taq II from Takara (Otsu, Japan). The relative expression levels were calculated using the ΔΔCt method[8]. ACTIN2/8 was used as an internal control. Primers used for this assay are listed in SupplementaryTable 1.

Supplementary Figures:

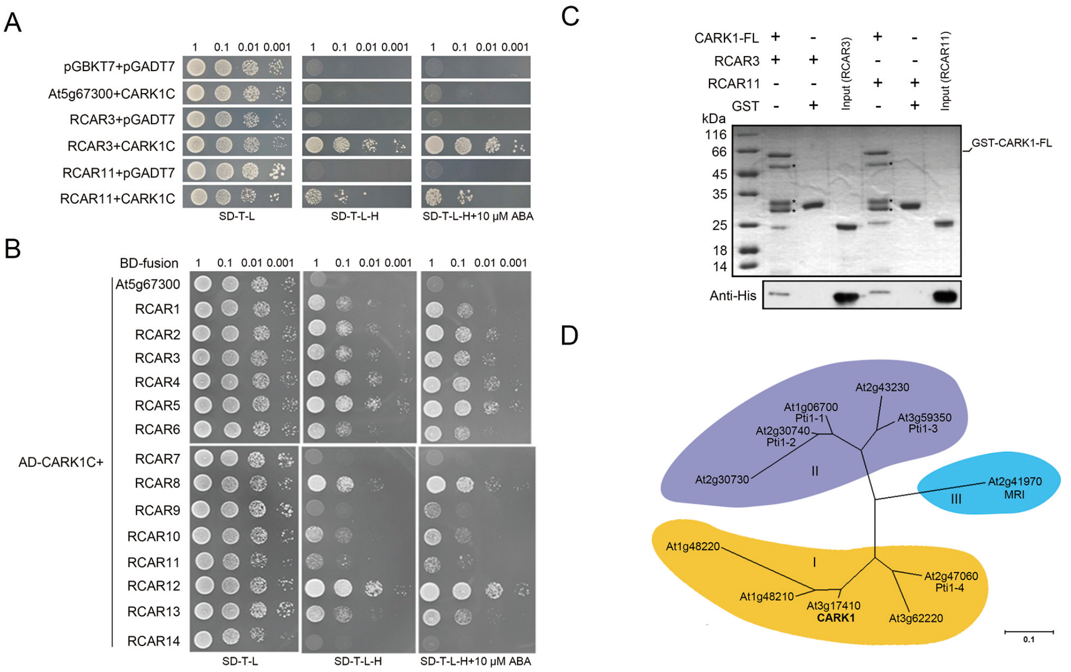

Supplementary Fig. S1 CARK1 interacts with RCARs in vitro. (**A**) CARK1 interacts with RCAR3 and RCAR11 in yeast. Dilutions (1, 0.1, 0.01 and 0.001) of saturated cultures were spotted onto the indicated plates. pGBKT7/pGADT7: empty bait/prey plasmid; At5g67300 (MYB44), an unrelated protein used as a negative control; CARK1C: a prey plasmid containing a truncated CARK1 (residues 290-364); RCAR3, RCAR11： the bait plasmids containing full-length RCAR3 or RCAR11. (**B**) The interaction between CARK1 and RCARs by Yeast Two-Hybrid Assay (Y2H). 14 members of RCAR family and the negative control At5g67300 (MYB44) fused with pGBKT7 vector and CARK1C (residues 290-364) fused with pGADT7 vector were examined in the assay as described in **A**. (**C**) GST pull-down assay using glutathione S-transferase (GST)-CARK1 (~66 kD) and 6×His-tagged RCAR3 (~22 kD) or RCAR11 (~23 kD) were performed with purified recombinant proteins as described in Fig. 3**a**. The top gel was visualized with Coomassie Blue staining, and bands labeled with asterisk were degradation products of GST fused CARK1 full-length; the bottom gel was the same sample order as the top one, anti-His antibody was used in the western blot analysis. (**D**) Phylogenetic analysis of the RLCK VIII family in Arabidopsis. Evolutionary analyses were conducted in MEGA5 using the neighbor-joining method.

A

B

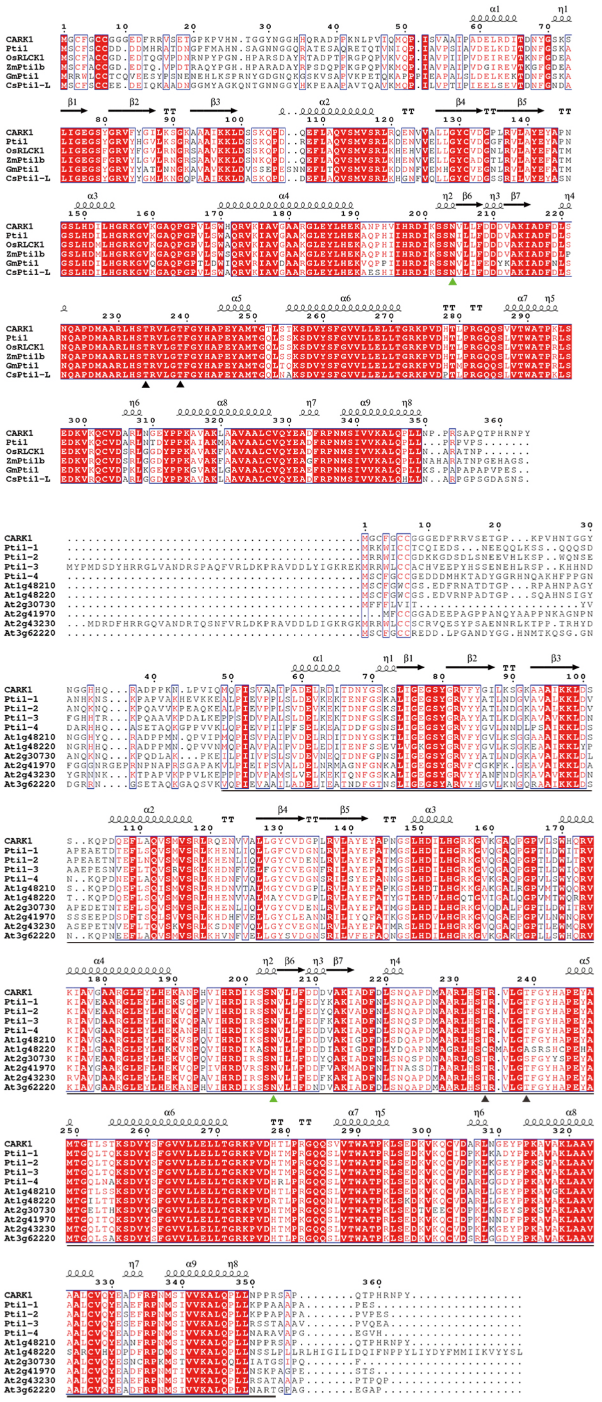

Supplementary Fig. S2 Sequence alignment of the CARK1 with its orthologous proteins. (**A**) The aligned sequences are as follows: CARK1 (Arabidopsis, NP_188367), Pti1 (Solanum lycopersicum, AAC61805), OsRLCK1 (Oryza sativa, NP_001049438), ZmPti1b (Zea mays, ABG36850), GmPti1 (Glycine max, AAO92595), and CsPti1 (Cucumis sativus, XP_004148576). (**B**) Sequence alignment of CARK1 and other 10 members of the RLCK VIII family in Arabidopsis. The sequence alignment is generated by ClustalW and the secondary structure of CARK1-KD is shown on top of the sequences using ESPript. Conserved residues are highlighted. The active site

(N204) and autophosphorylation sites (T234 and T239) are labeled with green triangle and black triangle under the bottom of the aligned sequences, respectively.

A

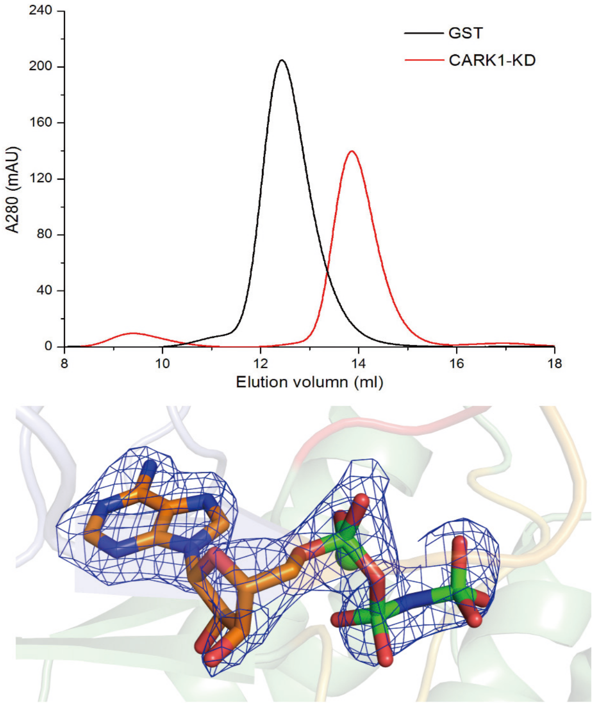

Supplementary Fig. S3 RCAR3 and RCAR11 interact with CARK1 in vitro. Size exclusion chromatography of CARK1-KD and Omit electron density map for CARK1-KD bound AMP-PNP molecule. (**A**) CARK1-KD (1mg) was injected onto a Superdex 75 10/300 GL column. The retention volume is 13.9 ml. Retention volume for the molecular weight standard (GST dimer, 52 kDa) is 12.5 ml. By comparison, we determined the monomeric state of CARK1-KD in solution. (**B**) The electron density map shown was omit map contoured at levels 2σ. AMP-PNP is shown with orange carbons, red oxygens, blue nitrogens, and green phosphorus atoms. The Mg ion is shown as green spheres.

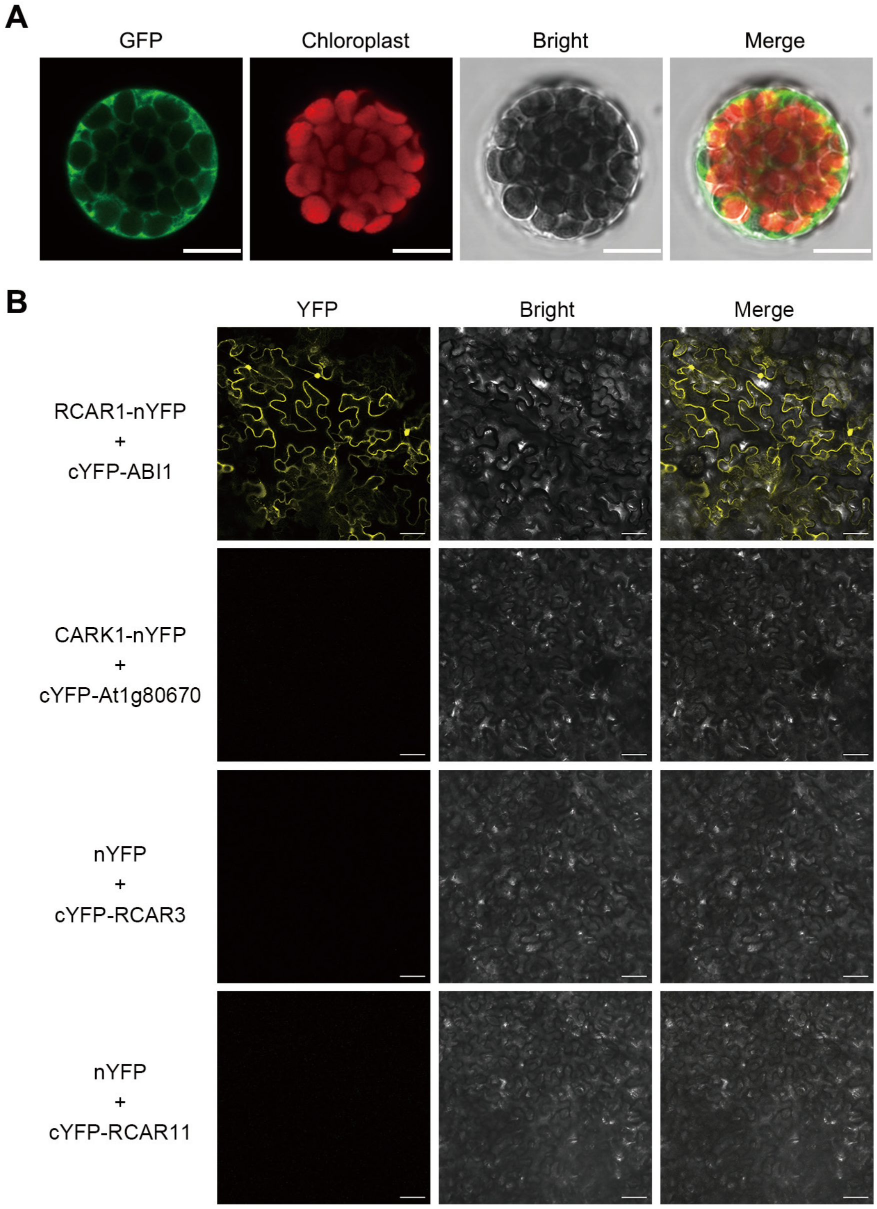

Supplementary Fig. S4 Subcellular localization of CARK1 and controls of BiFC assays. (**A**) Subcellular location of CARK1-GFP in Arabidopsis mesophyll protoplast. The 35S::CARK1-GFP construct was transfected into the protoplasts, and the GFP signal was detected using a fluoscence microscope after 16h transfection. Scale bars, 10 µm. (**B**) Co-expression of RCAR1-nYFP and cYFP-ABI1 (positive control) in leaves of *N. benthamiana*, yielded YFP signals both in the cytosol and in the nucleus. No signal of YFP fluorescence was detected after co-expression of cYFP-At1g80670 with CARK1-nYFP, nYFP with cYFP-RCAR3/RCAR11. At1g80670, one unrelated protein. Scale bar, 100 µm.

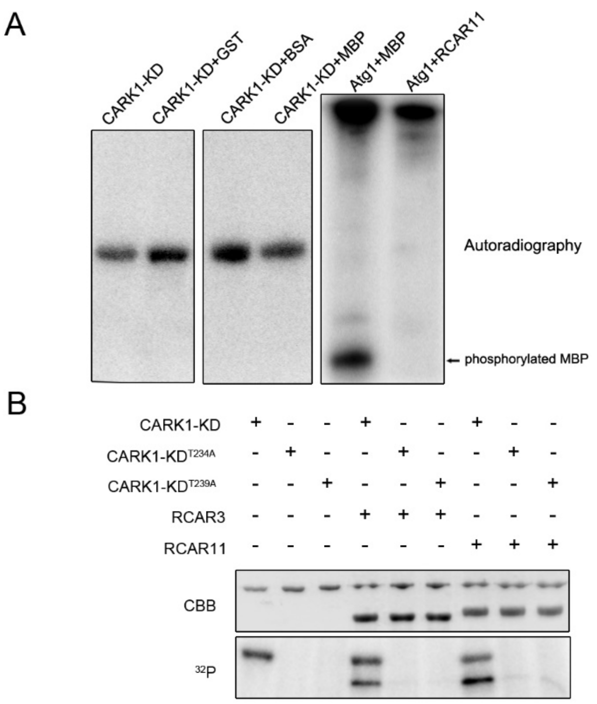

Supplementary Fig. S5 Controls of *in vitro* kinase assays and the effect of autophosphorylation activity of CARK1 on phosphorylation of RCAR3 and RCAR11. (**A**) Compared with phosphorylation of RCAR3 or RCAR11, no ^{32}P signal was detected after autoradiogram with incubation of CARK1-KD and GST or BSA, which was selected as negative controls. MBP, supposed to be the generic substrate of CARK1-KD, did not exhibit any phosphorylated band, consistent with previous studies of homologs (Pti1-1/3/4) in Arabidopsis. Meanwhile, tested RCAR11 could not be phosphorylated by another unrelated kinase, human Atg1 (which is a kinase required for autophage). As was shown, Atg1 can function normally by using MBP as its substrate. Arrow indicates the position of phosphorylated MBP. GST, glutathione S transferase. BSA, bovine serum albumin. MBP, myelin basic protein. (**B**) Compared with wild-type CARK1-KD, mutations at either residue T234 or T239 of CARK1-KD, which were previously defined as the key autophosphorylation sites in the activation loop of CARK1-KD, loss the kinase activity and could not phosphorylate RCAR3 or RCAR11. Top gel, Coomassie Brilliant Blue staining; Bottom gel, autoradiography. All reactions were performed as described in Fig. **3**.

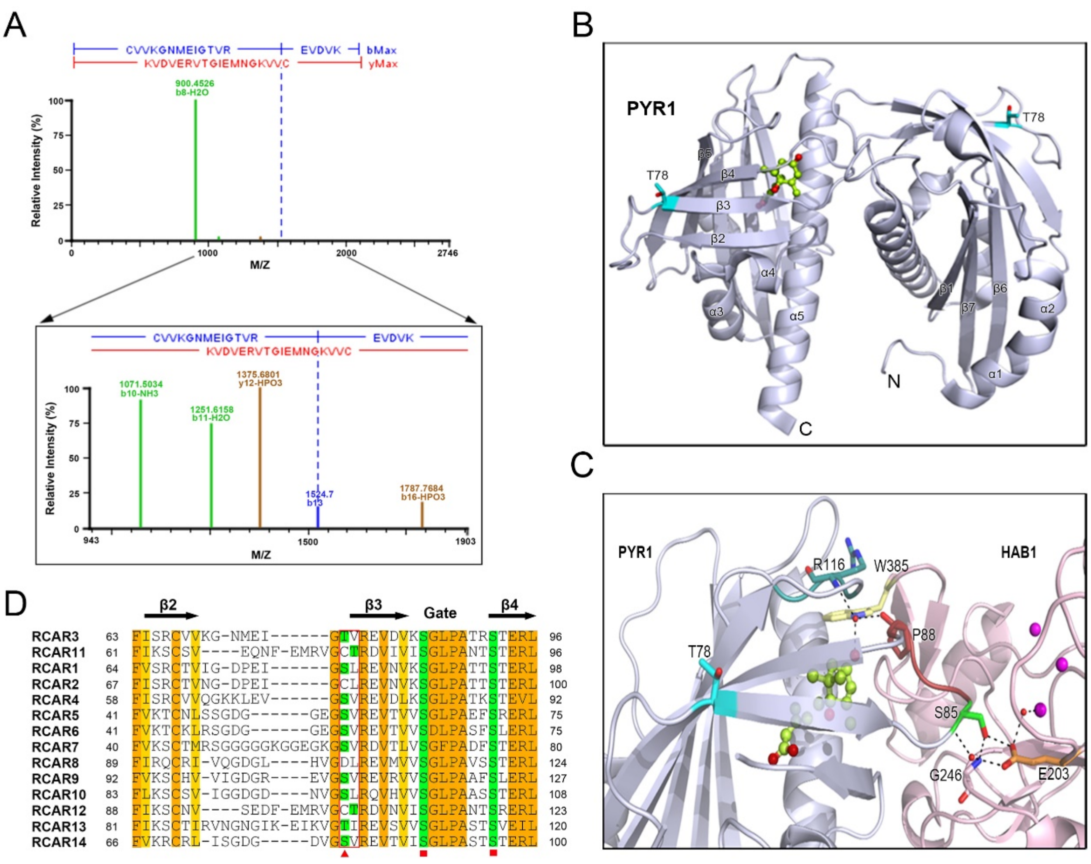

Supplementary Fig. S6 MS/MS spectra of the phosphopeptide from RCAR3 upon CARK1-KD treatment and Partial sequence alignment of RCAR family along with the structure of the PYR1 dimer and the PYR1-ABA-HAB1 complex in which the phosphorylation site is included. (**A**) Neutral loss of the phosphate group from the peptide precursor and particular fragment ions is strong evidence for the assignment of modification sites. Peptide sequence for individual spectrum is as follows: CVVKGNMEIG**pT**VREVDVK. (**B**) Structure of the dimeric PYR1 is colored by grey, the secondary structure elements and the N and C termini are indicated. Atoms of the bound (+)-ABA molecule are shown as limon spheres. (**C**) Structure of the PYR1-ABA-HAB1 complex. The PYR1 is colored by grey, the 'gate' loop containing S85 (green stick) and P88 and the 'latch' loop containing R116 are depicted by deep salmon and deep teal, respectively. The HAB1 catalytic domain is colored by light pink (Mn^{2+} ions as manga balls), the detail of interaction surface containing W385 (yellow), G246 (light pink) and E203 (orange) are indicated by sticks. The water molecules are shown as red spheres. Atoms of the phosphorylation residue T78 in (**B**) and (**C**) are highlighted by cyan sticks. (**D**) Partial sequence alignment of RCAR family with secondary structure elements of RCAR11 (PYR1) above the sequences and amino acid position of RCARs on the left and right column. Conserved residues are highlighted, phosphorylation sites identified by mass spectrum or predicted online (http://www.cbs.dtu.dk/services/NetPhos/) are

colored by green and labeled with red triangle or red square below the sequence, respectively. Gate residues are noted.

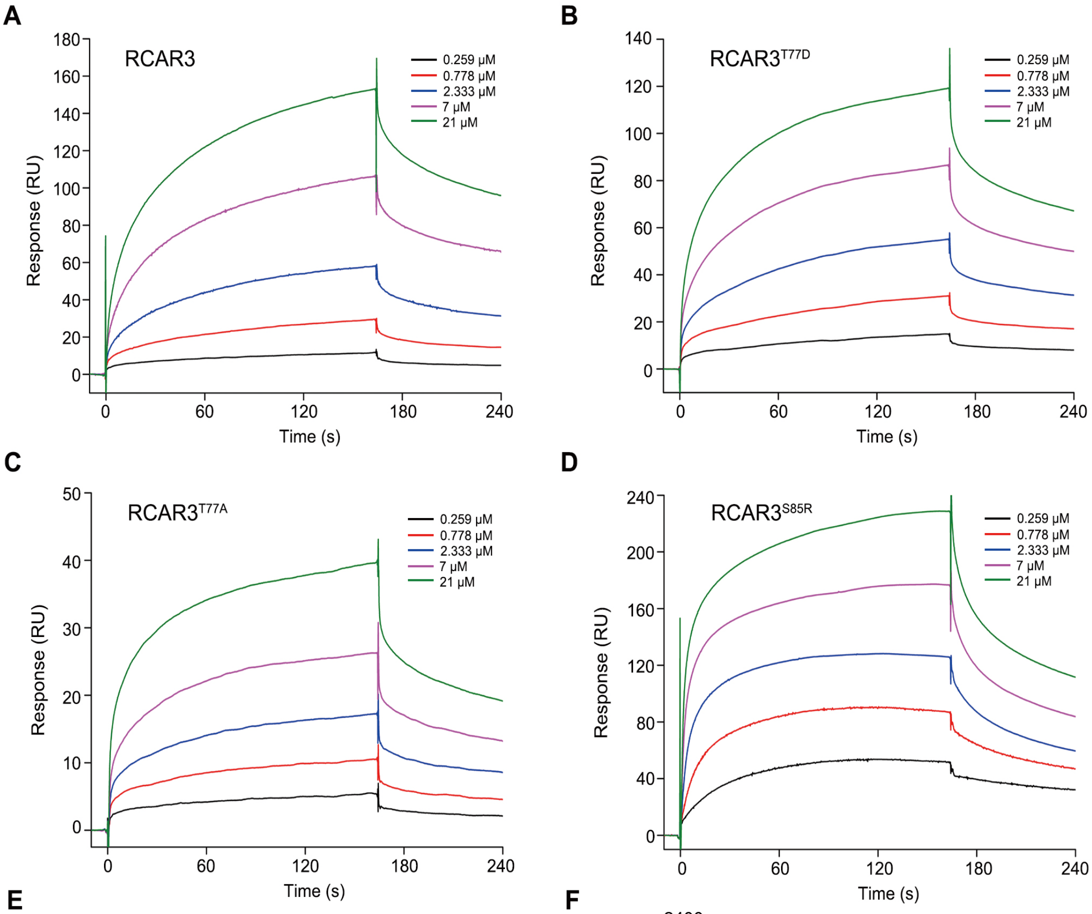

E

Analyte	ka ($M^{-1}S^{-1}$)	kd (S^{-1})	Kd (M)	χ^2
RCAR3	936.6	8.220×10^{-4}	8.776×10^{-7}	3.57
RCAR3^{T77D}	4441	8.675×10^{-4}	1.953×10^{-7}	8.02
RCAR3^{T77A}	721.9	4.605×10^{-3}	6.380×10^{-6}	0.949
RCAR3^{S85R}	600.9	6.759×10^{-3}	1.125×10^{-5}	34.5

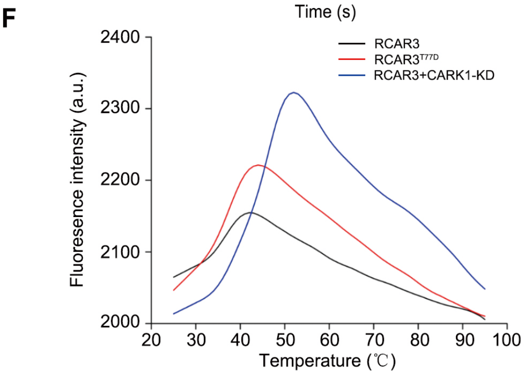

Supplementary Fig. S7 Surface Plasmon Resonance (SPR) assay for RCAR3 variants and ABI1 and Effect of phosphorylation on RCAR3 thermal stability. The interactions between RCAR3 variants and ABI1 were measured with SPR. Purified ABI1 was immobilized on a CM5 sensor chip followed by the manufacturer's manual. Wild type RCAR3 (**A**) and its variants, including RCAR3^{T77D} (phosphor-mimic form) (**B**), RCAR3^{T77A} (phosphor-defective form) (**C**), RCAR3^{S85R} (interface point mutant) (**D**), were injected over the immobilized ABI1-sensor chip. Injection concentrations of analytes were serially diluted by 3-fold: 0.259μM, 0.778 μM, 2.333μM, 7μM and 21μM. The kinetic parameters of ka ($M^{-1}s^{-1}$), kd (s^{-1}) and Kd (M, dissociation constant) were calculated based on the SPR experiments (**E**). (**F**) Representative melting curves of RCAR3 and its variants thermal stability in HEPES buffer (20 mM HEPES pH 7.5, 100 mM NaCl) measured by differential scanning

fluorimetry (DSF) assay. Calculated T_m values are: RCAR3, 36 ℃; RCAR3^{T77D}, 38 ℃; pretreated RCAR3 (RCAR3 and CARK1-KD at the molar ratio of 10:1), 43 ℃.

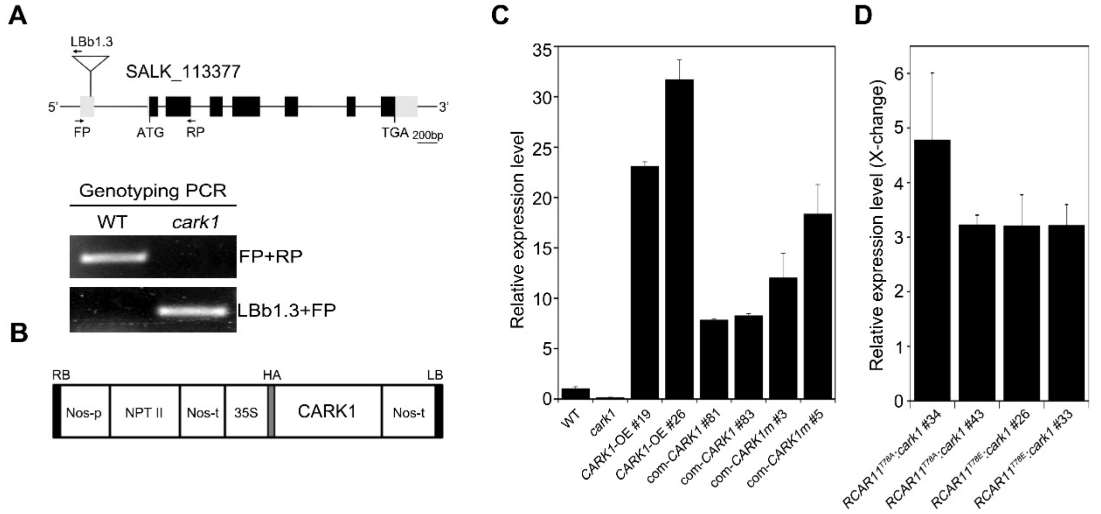

Supplementary Fig. S8 Identification of T-DNA insertion mutant plants (*cark1*), *CARK1* overexpression lines (*CARK1-OEs*) and transgenic lines of phosphor-mimic and phosphor-deficient RCAR11. (**A**) Schematic representation of the *cark1* mutant with T-DNA insertion, and genotyping PCR of *cark1*. Dark boxes represent exon, lines between dark boxes indicate introns, and gray boxes represent 5'- and 3'-untranslated regions. T-DNA insertion site is indicated by an inverted triangle. T-DNA-specific (LBb1.3) and gene-specific (FP and RP) primers used in genotyping PCR are marked by arrows. (**B**) Construct used for plant. RB, right T-DNA border; LB, left T-DNA border; NOS-p, NOS promoter; NOS-t, NOS terminator; 35S, cauliflower mosaic virus (CaMV) 35S promoter; NPTII, neomycin phosphotransferase II; HA, influenza hemagglutinin. (**C**) Real time qRT-PCR analysis of the wild type, *cark1* mutant, *CARK1*-OE lines and *CARK1*/*CARK1m* complemented lines (com-*CARK1* and com-*CARK1m*). Expression levels of *CARK1* transcripts were determined by qRT-PCR using gene-specific primers. ACTIN2/8 mRNA levels were used as a loading control. (**D**) Relative expression levels of RCAR11 in the wild-type and *RCAR11^{T78A}:cark1*, *RCAR11^{T78E}:cark1* under 50 μM ABA treatment and without ABA. The *RCAR11* expression without ABA treatment was set to 1, and analysis of *RCAR11* expression levels changes in the absence and presence of ABA.

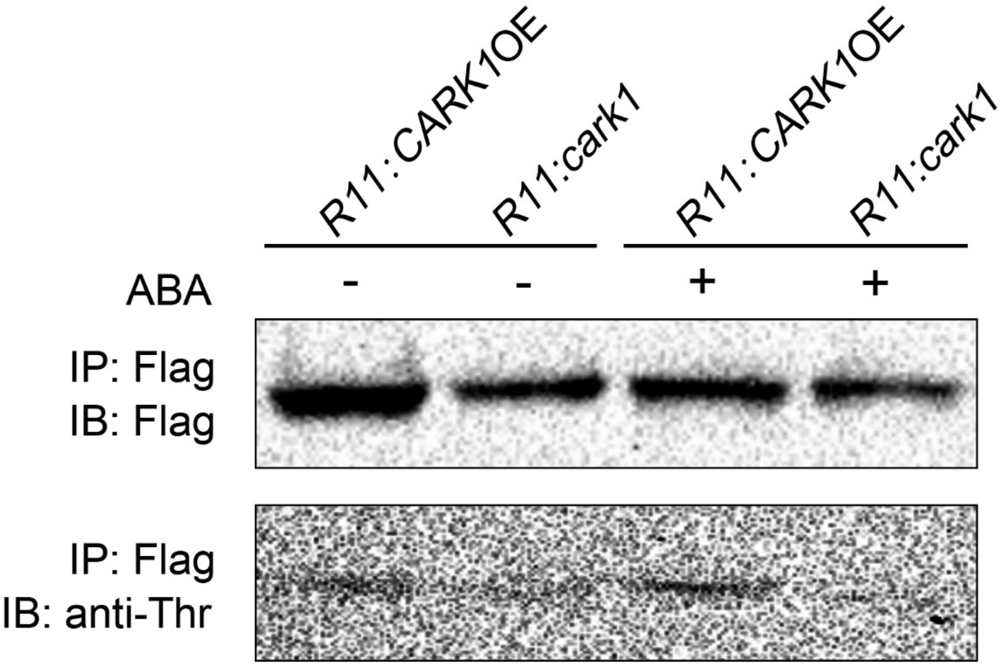

Supplementary Fig. S9 Phosphorylation status of endogenous RCAR11 in transgenic plants with or without 50 μM ABA treatment for 1 h. For more details of the assay, see the legend of Fig. 3g. Transgenic lines of *R11:CARK1*OE and *R11:cark1* used in this assay were #3 and #3, respectively.

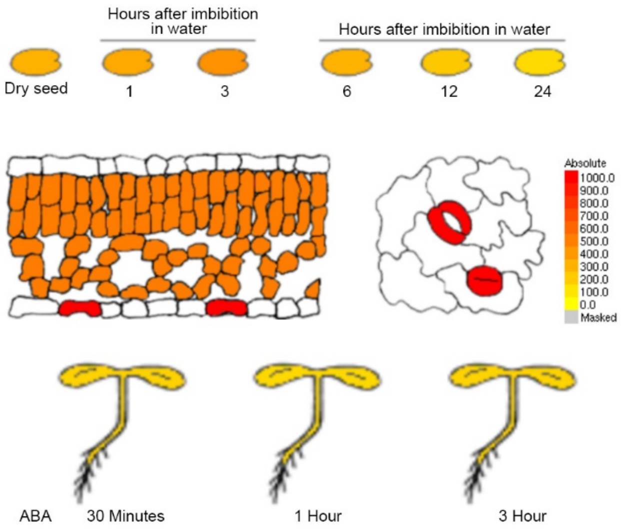

Supplementary Fig. S10 Gene expression patterns of CARK1. The expression data for CARK1 were collected from a public database, eFP browser (http://bbc.botany.utoronto.ca/efp/cgi-bin/efpWeb.cgi). This Fig. displays a part of the data from 'Seed', 'GC (guard cell)', and 'Hormone' categories. The signal threshold was manually adjusted to 1,000.

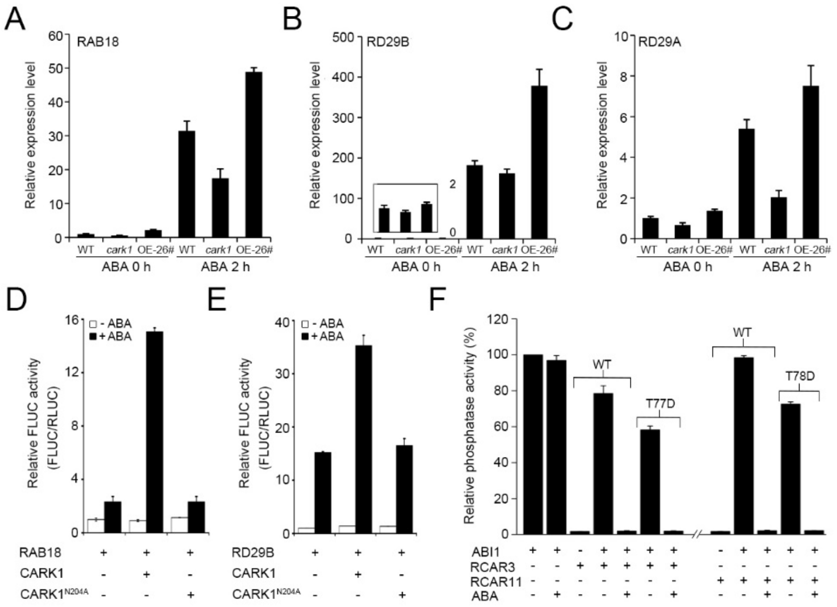

Supplementary Fig. S11 CARK1 positively regulates ABA signaling pathway. (**A-C**) CARK1 positively regulate the expression of ABA-regulated genes. Real-time qRT-PCR was used to analyze the expression of ABA-regulated *RAB18* (**A**), *RD29B* (**B**) and *RD29A* (**C**) in Arabidopsis. 10 μM ABA was used. ACTIN2/8: the internal control. Col, wild type. *cark1*, mutant. OE-26#, *CARK1* overexpressing line 26. Error bars indicate SD (n=3). (**D** and **E**) Relative reporter activity (FLUC/RLUC) of ABA-responsive gene *RAB18* (**D**) and *RD29B* (**E**) expression in *cark1* mutant. The dual-LUC plasmid bearing the reporter (*35S::RLUC*) and the effector (p*RAB18::FLUC* and p*RD29B::FLUC*) was transfected into *cark1* protoplasts in the presence or absence of ABA. Open bars: without ABA. Filled bars: 10 μM ABA. *RLUC*: the internal control. Error bars represent SD (n=3). (**F**) Effects of various phosphorylation forms of RCAR3/RCAR11 on the inhibition of ABI1 activity in the absence or presence of ABA. The concentrations of each protein component were 0.3 μM for ABI1, 3 μM for WT and phosphor-mutants of RCAR3/RCAR11, and 10 μM (+)-ABA. Error bars represent SD (n=3).

Supplementary Table 1

Primers used in this study

Assays	Genes	Primer sequences (5'–3')
Yeast two-hybrid assay	RCAR3	F (*EcoR*I): CCG*GAATTC*ATGGAAGCTAACGGGATTGAGAAC
		R (*Pst*I): AA*CTGCAG*TTAGACTCTCGATTCTGTCGTGTCTTG
	RCAR11	F (*EcoR*I): CCG*GAATTC*ATGCCTTCGGAGTTAACACC
		R (*BamH*I): CGC*GGATCC*TCACGTCACCTGAGAACCACTT
	CARK1C	F (*EcoR*I): CCG*GAATTC*TGGGCAACCCCTAAATTGAGTGAAG
		R (B*amH*I): CGC*GGATCC*TCAATACGGGTTCCTGTGTGGAG
Genotyping PCR analysis	CARK1	F: TTTCTTCACCAACCCTACACG
		R: CAACAGAGTGACTTCGTGCAG
	T-DNA	LB1.3b: ATTTTGCCGATTTCGGAAC
Transgenic plant construction	CARK1	F (*Xba*I): GC*TCTAGA*ATGGAGTACCCATACGACGTACATGGGCTGCTTTGGTTGTTG
		R (*Sma*I): TCC*CCCGGG*TCAATACGGGTTCCTGTGTG
qRT-PCR analysis	CARK1	F: GAATTTCTCGCCCAGGTATCA
		R: GTCCTCTAGCCGCACCAACA
	RCAR11	F: ATCGTCATCAGTGGATTA
		R: TAATTCGTCAGCCTATGT
	RAB18	F: ATGGCGTCTTACCAGAACCGT
		R: CCAGATCCGGAGCGGTGAAGC
	RD29B	F: ACTGATCCCACGCATAAAGG
		R: TCATTCTACCAAGAGACTCAGCA
	RD29A	F: ACGTCGAGACCCCGATAAC
		R: CAATCTCCGGTACTCCTCCA
	ACTIN2/8	F: GGTAACATTGTGCTCAGTGGTGG
		R: AACGACCTTAATCTTCATGCTGC
Transient activation assay	RAB18 promoter	F: CCCAAGCTTGCTAATTAGTAAGTGATTTAC
		R: CGGGATCCACTTTGAGCTAAGC
	RD29B promoter	F: CCCAAGCTTTCCGTTGTAGACGATGGGTGC
		R: CGGGATCCTCAAGTGAATCAATC
BiFC vector construction	CARK1-nYFP	F (*Kpn*1): CGG*GGTACC*ATGGGCTGCTTTGGTTGTTGTG

		R (*MluI*): CG*ACGCGT*CATACGGGTTCCTGTGTGGAGTCT
	CARK1^{N204A}-nYFP	F (*Kpn*1): CGG*GGTACC*ATGGGCTGCTTTGGTTGTTGTG
		R (*Mlu*I): CG*ACGCGT*CATACGGGTTCCTGTGTGGAGTCT
	cYFP-RCAR3	F (*Kpn*1): CGG*GGTACC*ATGGAAGCTAACGGGATTGAGAAC
		R (*Sal*I): ACGC*GTCGAC*TTAGACTCTCGATTCTGTCGTGTCT
	cYFP-RCAR11	F (*Kpn*1): CGG*GGTACC*ATGCCTTCGGAGTTAACACCAG
		R (*Sal*I): ACGC*GTCGAC*TCACGTCACCTGAGAACCACT
	cYFP-At1g80670	F (*Kpn*1): CGG*GGTACC*ATGGCAACTTTTGGTGCGCCTG
		R (*Sal*I): ACGC*GTCGAC*TCATTTTCTGCCGGTTGCTCCAACTCT
Heterologous expression vector construction	pET28a-CARK1-KD	F (*Nco*I): CATG*CCATGG*CGCATCATCATCATCATCATGACGGCGT TGAAGGCGGCA
		R (*Hind*III): CCC*AAGCTT*TCAACGAGGAGGATTGAGCAACGGC
	pET28a-RCAR3	F (*Nco*I): CATG*CCATGG*AAGAAGCTAACGGGATTGAGAACT
		R (*Xho*I): CCG*CTCGAG*GACTCTCGATTCTGTCGTGTCTTGA
	pET28a-RCAR11	F (*Nco*I): CATG*CCATGG*CGCCTTCGGAGTTAACACCAGAAG
		R (*Xho*I): CCG*CTCGAG*CGTCACCTGAGAACCACTTCCGTCA
	pGEX6p-CARK1-KD	F (*BamHI*): CGC*GGATCC*CAGCCTATCTCTGTTGCG
		R (*Hind*III): CCC*AAGCTT*TCAACGAGGAGGATTGAGCAACGGC
	pGEX6p-RCAR3	F (*BamHI*): CGC*GGATCC*ATGGAAGCTAACGGGATTGAG
		R (*Hind*III): CCC*AAGCTT*TTAGACTCTCGATTCTGTCGTGTCTTGA
	pGEX6p-RCAR11	F (*BamHI*): CGC*GGATCC*ATGCCTTCGGAGTTAACACCAG
		R (*Xho*I): CCG*CTCGAG*TCACGTCACCTGAGAACCACT
PP2C phosphatase assay	ABI1	F (*BamHI*): CGC*GGATCC*AGTAGGAGTTTGTTTGAATTC
		R (*Xho*I): CCG*CTCGAG*TCAGTTCAAGGGTTTGCTCT

Supplementary Table 2

Statistics of data sets and structure refinement

Parameters	Native	Se-MAD peak
**Data collection statistics**		
Cell parameters		
a (Å)	69.715	46.787
b (Å)	46.661	99.787
c (Å)	110.512	153.281
α, β, γ (°)	90.0, 105.8, 90.0	90.0, 90.0, 90.0
Space group	$P2_1$	$P2_12_12_1$
Wavelength used (Å)	1.0000	0.9789
Resolution (Å)	50.0–1.9 (1.93-1.90) [c]	50.0–2.4 (2.45-2.40)
Total/Unique reflections	209,723 (103,130)	431,169 (32,835)
Completeness (%)	97.7 (84.9)	96.6 (93.5)
Average I/σ(I)	15.0 (7.7)	7.8 (3.1)
R_{merge} [a] (%)	5.5 (59.6)	11.1 (64.5)
CC1/2 for highest resolution (%) [d]	84.7	82.3
**Refinement statistics**		
No. of reflections used (σ(F) > 0)	53,479	
R_{work} [b] (%)	21.68	
R_{free} [b] (%)	25.12	
r.m.s.d. bond distance (Å)	0.009	
r.m.s.d. bond angle (°)	1.263	
Average B-value (Å^2)		
Average B-value for protein atoms	36.3	
Average B-value for ligand atoms	30.4	
Average B-value for solvent atoms	45.5	
No. of atoms		

No. of protein atoms	4,341
No. of ligand atoms	64
No. of solvent atoms	363
Ramachandran plot	
Res. in favored regions (%)	98.55
Res. in allowed regions (%)	1.45
Res. in outlier regions (%)	0.00

[a] $R_{merge} = \Sigma_h\Sigma_l \mid I_{ih}-\langle I_h\rangle \mid / \Sigma_h\Sigma_I \langle I_h\rangle$, where $\langle I_h\rangle$ is the mean of the observations I_{ih} of reflection h.

[b] $R_{work} = \Sigma(||F_p(obs)|-|F_p(calc)||)/ \Sigma|F_p(obs)|$; R_{free} is an R factor for a pre-selected subset (5%) of reflections that was not included in refinement.

[c] Numbers in parentheses are corresponding values for the highest resolution shell.

[d] CC1/2 was calculated by HKL2000.

References

1. Adams, P. D. *et al*. PHENIX: a comprehensive Python-based system for macromolecular structure solution. *Acta Crystallogr. D* **66**, 213-221 (2010).
2. Emsley, P., & Cowtan, K. Coot: model-building tools for molecular graphics. *Acta Crystallogr. D* **60**, 2126-2132 (2004).
3. Yoo, S. D., Cho, Y. H. & Sheen, J. Arabidopsis mesophyll protoplasts: a versatile cell system for transient gene expression analysis. *Nat. Protoc.* **2**, 1565-1572 (2007).
4. Song, S. *et al*. The Jasmonate-ZIM domain proteins interact with the R2R3-MYB transcription factors MYB21 and MYB24 to affect Jasmonate-regulated stamen development in Arabidopsis. *Plant Cell* **23**, 1000-1013 (2011).
5. Yin, P. *et al*. Structural insights into the mechanism of abscisic acid signaling by PYL proteins. *Nat. Struct. Mol. Biol.* **16**, 1230-1236 (2009).
6. Clough, S. J. & Bent, A. F. Floral dip: a simplified method for Agrobacterium-mediated transformation of Arabidopsis thaliana. *Plant J.* **16**, 735-743 (1998).
7. Hellens, R. P. et al. Transient expression vectors for functional genomics, quantification of promoter activity and RNA silencing in plants. *Plant methods* **1**, 13 (2005).
8. Livak, K. J. & Schmittgen, T. D. Analysis of relative gene expression data using real-time quantitative PCR and the 2(-Delta Delta C(T)) Method. *Methods* **25**, 402-408 (2001).
